# Role of the exercise professional in metabolic and bariatric surgery

**DOI:** 10.1016/j.soard.2023.09.026

**Published:** 2023-09-27

**Authors:** Matthew A. Stults-Kolehmainen, Dale S. Bond, Laura A. Richardson, Louisa Y. Herring, Bethany Mulone, Carol Ewing Garber, John Morton, Saber Ghiassi, Andrew J. Duffy, Ethan Balk, Charles J. Abolt, Matt C. Howard, Garrett I. Ash, Susannah Williamson, Emilian Rejane Marcon, Melissa De Los Santos, Samantha Bond, Janet Huehls, Osama Alowaish, Nina Brojan Heyman, Bruno Gualano

**Affiliations:** a Division of Digestive Health, Center for Weight Management, Yale New Haven Hospital, New Haven, Connecticut; b Department of Biobehavioral Sciences, Teachers College – Columbia University, New York, New York; c Departments of Surgery and Research, Hartford Hospital/HealthCare, Hartford, Connecticut; d School of Kinesiology, University of Michigan, Ann Arbor, Michigan; e University Hospitals of Leicester NHS Trust, Infirmary Square, Leicester, England, UK; f NIHR Leicester Biomedical Research Centre, Leicester Diabetes Centre, Leicester General Hospital, Leicester, England, UK; g Division of Bariatric and Minimally Invasive Surgery, Department of Surgery, Yale University School of Medicine, New Haven, Connecticut; h Department of Nutrition and Food Studies, New York University, New York, New York; i Earth and Environmental Sciences Division, Los Alamos National Laboratory, Los Alamos, New Mexico; j Department of Marketing & Quantitative Methods, University of South Alabama, Mobile, Alabama; k Center for Medical Informatics, Yale School of Medicine, New Haven, Connecticut; l Center for Pain, Research, Informatics, Medical Comorbidities and Education Center (PRIME), VA Connecticut Healthcare System, West Haven, Connecticut; m Center for Military Psychiatry and Neuroscience, Walter Reed Army Institute of Research (WRAIR), Silver Spring, Maryland; n Army Educational Outreach Program, Rochester Institute of Technology, Rochester, New York; o Department of Bariatric Surgery, Hospital de Clínicas de Porto Alegre, Porto Alegre, Brazil; p College of Applied Health Sciences, University of Illinois at Chicago, Chicago, Illinois; q UMass Memorial Weight Center, UMass Memorial Medical Center, Worchester, Massachusetts; r Applied Physiology and Nutrition Research Group - Center of Lifestyle Medicine, Faculdade de Medicina FMUSP, Universidade de São Paulo, São Paulo, Brazil

**Keywords:** Metabolic and bariatric surgery, Physical activity, Implementation, Exercise prescription, Mixed methods, Qualitative

## Abstract

**Background:**

Physical activity (PA) is important for the long-term health and weight management of patients who undergo metabolic and bariatric surgery (MBS). However, the roles of exercise professionals in MBS settings have not been systematically determined.

**Objectives:**

To investigate: (1) who are the professionals implementing PA programming in MBS clinical settings; and (2) what patient-centric tasks do they perform?

**Setting:**

Clinical and academic exercise settings worldwide.

**Methods:**

This multimethod study included a scoping review of PA programs in MBS described in the research literature. Data about job tasks were extracted and provided to 10 experts to sort into categories. Cluster analysis was utilized to find the hierarchical structure of tasks. A Delphi process was used to agree on a final model.

**Results:**

The majority of PA professionals were exercise physiologists in the USA and physiotherapists or other types of exercise professionals elsewhere. Forty-three tasks were identified, the most reported being supervision of exercise, fitness testing, and exercise prescription. Seven higher-order categories were determined: (1) Exercise-related health assessment, (2) Body composition and physical fitness assessment, (3) Lifestyle physical activity and sedentary behavior assessment, (4) Education, instruction, and prescription, (5) Exercise monitoring, (6) Behavioral counseling and psychosocial support, and (7) Dietary support. The following statements were rated an average of 9.0, classifying them as “imperative”: 1) “Pre- and postoperative PA/exercise guidelines for MBS patients are needed”, 2) “MBS programs need to include PA/exercise as part of multidisciplinary care”.

**Conclusions:**

The expert group reached a consensus on 7 major classifications of job tasks for the exercise professional. It is important for governing medical associations across the world to formally recognize experienced exercise professionals as playing pivotal roles in continuing, multidisciplinary care for MBS patients. These findings also provide evidence-based information in the effort to solidify these positions within the greater context of healthcare.

Metabolic and bariatric surgery (MBS) yields profound weight loss and health improvements, although there is substantial variability in the amount and trajectories of these outcomes [1]. Some of this variability may be attributed to the physical activity (PA) behaviors of patients who had MBS. In this population, more moderate-to-vigorous intensity PA, less sedentary behavior, and more steps are associated with greater weight loss, better weight maintenance, better bone health, and improvements in cardiometabolic health parameters [2–6]. However, patients overall do not become spontaneously active after surgery. Indeed, an average patient is insufficiently active before surgery and makes only small changes in activity behaviors after surgery, thus remaining insufficiently active [7]. In the absence of exercise, several important benefits of MBS (e.g., improvements in insulin resistance, inflammatory markers, endothelial function) are reversed, even in a short period of time, as has been demonstrated in a recent trial [8].

The difficulties that patients experience in making substantial changes in their activity behaviors suggest that they need additional information, intervention, and support to make such changes [9]. Indeed, structured exercise and PA programming in combination with dietary counseling may help to optimize and sustain MBS outcomes [4–6,10]. Unfortunately, it is likely that such programs are infrequently provided in real-world, clinical settings [9,11]. Likewise, there is some data suggesting that even simple exercise guidance is not routinely provided in MBS health services worldwide [9,12]. This may partly be the case because, while established exercise guidelines currently exist for hypertension, diabetes, and other chronic conditions [13], there are no specific guidelines for exercise with MBS. Furthermore, effectively translating exercise programs from clinical trials into clinical practice is difficult to achieve [3,14–16]. Despite these shortcomings, insurance companies often require the provision of exercise counseling as a condition for surgical approval [17]. For instance, the current Aetna policy refers to the exercise physiologist as one professional qualified to supervise a ”multi-component behavioral intervention program” [18].

In an effort to translate physical activity research into clinical practice, Koorts and colleagues [19] developed the PRACTIS Model. According to this framework, several major considerations must be initially targeted, including the clinical population, context/place, intervention characteristics and the people conducting the implementation – the interventionist. While the first parts have been widely discussed [20], the last component has been infrequently addressed in the medical literature [16,21,22]. Key questions surrounding exercise personnel include: a) who delivers the intervention, b) what skills, education, and qualifications should they hold, and c) how are they trained and supported? While this critical component of the PRACTIS Model has been addressed in primary care [22,23], it has not received adequate attention in the context of MBS. Typically, this is not the physician or surgeon, who usually lacks the time and training [24], but it may include an exercise professional (EP) working as part of a multidisciplinary team [25].

Exercise professionals comprise a large and diverse group of individuals all aiming to improve the health of patients through physical activity [26]. Unfortunately, only recently have guidelines been established to report this information in the scholarly literature [27], and researchers are just beginning to conform to this initiative [28]. To fill these important knowledge gaps, the major objective of the current study was to utilize a rigorous multimethod approach [29], including a scoping review and thematic analysis, to answer 2 main questions: 1) Who are the exercise professionals conducting PA programming in MBS? (e.g., exercise physiologists versus physiotherapists/physical therapists), and 2) What are the prominent clinical exercise job tasks conducted by EPs in an MBS setting? In this effort, we secondarily aimed to: a) describe the qualifications, training, and/or background of the professionals who are delivering the interventions, and b) determine if the skills of the EP vary by field. Lastly, we aimed to address issues pertinent to the implementation of PA programming in MBS (e.g., guidelines, reimbursement).

## Methods

**Fig. 1.**
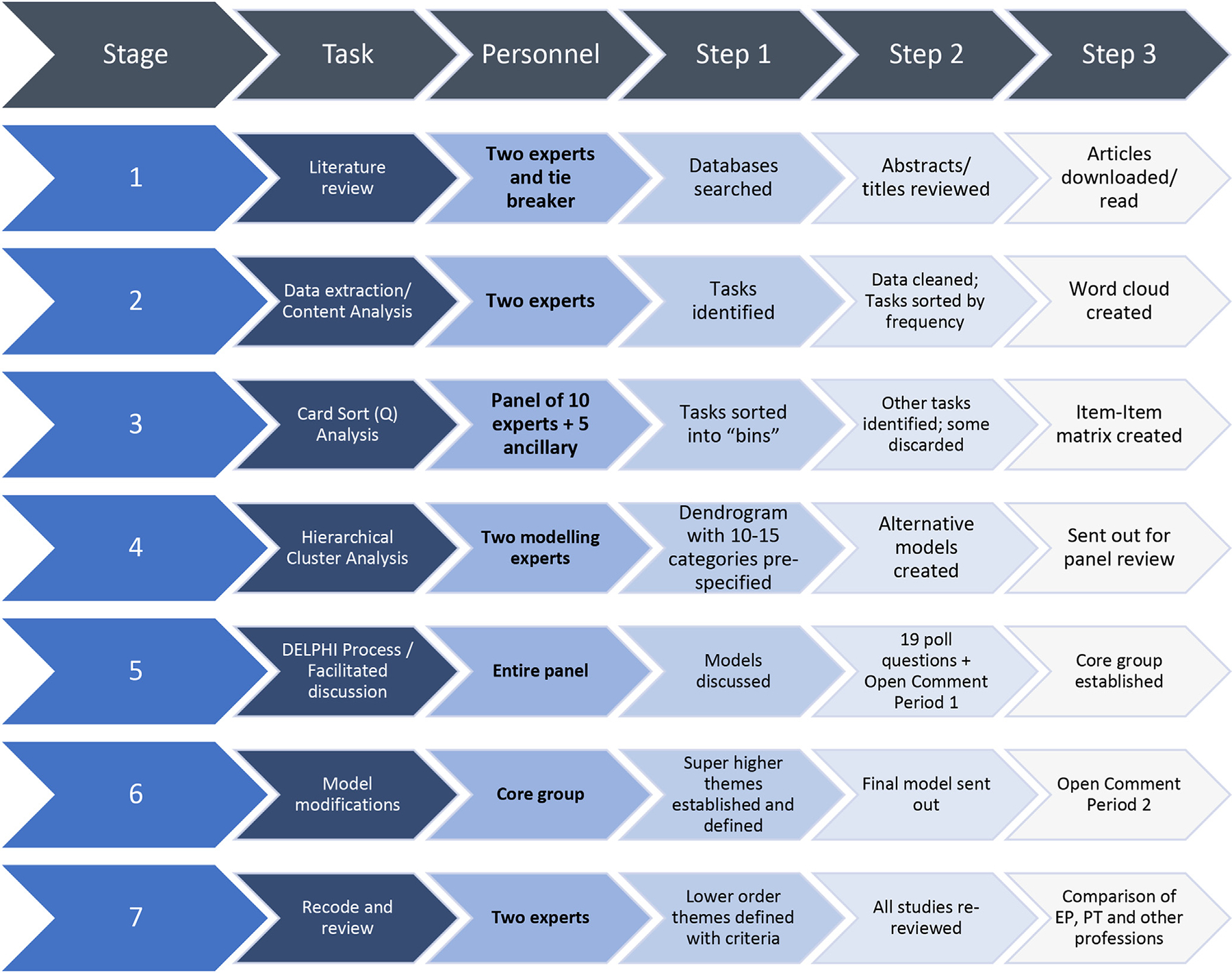
Systematic Clinical/Organizational Role Evaluation (SCORE) technique used in the current study.

To appropriately address the research questions, we utilized a blend of qualitative and quantitative methods – The Systematic Clinical/Organizational Role Evaluation (SCORE) technique, which integrates: the AGREE model [30], inductive thematic analysis [31], Card sort (Q) analysis [32], Hierarchical cluster analysis [33], and the Delphi process [34]. The AGREE Model for clinical decision making, which has been used to develop other clinical exercise protocols, like the Physical Activity Readiness-Questionnaire (PAR-Q) [35], was modified for the current task. See Figure 1.

### Literature review

Per the AGREE model [30], we started with a scoping review of the literature (Stage 1) that took place between June, 2021 and January, 2022 in the Web of Science (Core Collection) and PubMed databases. PRISMA guidelines were followed [36]. The review included peri-operative MBS literature from the United States and globally to capture a wider lens of therapeutic exercise interventions. Titles and abstracts were searched, with the first term being “bariatric” and the second term being “exercise” or “physical activity.” Database results were filtered by language (English and Portuguese only), use of human subjects and studies publishing original data (e.g., eliminating comments, reviews, guidelines, editorials). Duplicate studies were then eliminated. During screening of titles and abstracts, studies were required to describe exercise and PA training and/or education programs (e.g., not just observe participants’ PA over time). Refer to Supplement 1. Some articles were obtained by scanning the following: a) review papers, b) personal archives (i.e., relevant articles already in the authors’ possession), c) reference lists from all studies obtained, and d) Google Scholar alerts for “Bariatric” and “exercise.” Full text articles were obtained and read for eligibility. See the PRISMA checklist in Supplement 2.

### Data extraction

Two investigators (MSK and MD) extracted data related to patient-centered (clinical) exercise and physical activity program tasks completed by the exercise professional (Stage 2). An initial list of job tasks was identified through methods described in Supplement 3.

### Thematic and card sort (Q) analysis

In the third stage, these data were categorized with a mixed inductive thematic analysis [29] and card sort (Q) analysis [32]. Data were systematically reduced by MSK and MD, who conducted cleaning and recoding. This included nominalization of verbs. Data were examined as follows: a) first as multiple words (e.g., “heart rate monitoring”) and then, b) connected/adjoined with a hyphen (e.g., “HR-monitoring”) to facilitate visualization. Tasks were then compared for overlap and agreement between investigators. Unique job tasks were entered into a free word cloud program (freewordcloudgenerator.com, Salt Lake City, UT) to visually inspect (black background, rainbow color pattern font), categorize by word frequency, and provide labels [37]. See Supplement 4.

An expert group of bariatric clinicians, exercise practitioners, and scientists were invited to complete the sort task. The Delphi process requires 8–10 people, but due to final exams and summer holidays, we did not expect that all people would respond. Therefore, we over-sampled (15) so at least 8 people would be involved at each stage. Ten had complete and valid responses submitted on time. The remainder did not respond in the required timeframe (3), had incomplete responses (1) or completed the form erroneously (1). Analysts included 4 exercise physiologists, 2 registered dietitians one advanced practice registered nurse, one surgeon, and 2 exercise scientists). Four were exercise practitioners in MBS clinics, each with a minimum of 9 years of practice in the area, for a total of 66 years (MSK, LR, JH, RM). Those not responding at this stage continued to follow the process, evaluate data, and more actively participate in later stages. Individuals in the expert group independently inspected the data, identified related tasks and sorted/categorized these into “bins”/clusters starting with the most frequent job tasks observed. To prevent author word choice from hiding findings (e.g., “body composition testing” versus “analysis of body composition”), high frequency words were collapsed with similar words and then re-analyzed. The initial goal was 10–15 bins or clusters, based on Hsieh & Shannon [31]. Respondents could elect to discard items or suggest new ones. Responses from these 10 experts were included into an item-by-item matrix, which was used to determine distance of each item from the others [32].

### Hierarchical cluster analysis (HCA)

In Stage 4, a hierarchical cluster analysis was then utilized (Matlab v R2022a) [33] to create dendrogram models based on distance between word items. Two alternative dendrogram models were generated for group consideration. Both lower-order and higher-order themes (HOTs) were detected from the dendrograms. To identify higher-order themes (to generate the a priori, 10–15 themes) a distance threshold was set to 7. To identify 7 super higher-order themes (SHOTs), a distance threshold was set to 9.

### Delphi process: issues in bariatric exercise survey

In Stage 5, a process of reaching consensus among the expert group was initiated to determine a final list of job task categories and to address related issues, based on the Delphi Technique [34]. This included 2 group discussion sessions (both in June, 2022) to: a) review the HCA, b) consider adding, deleting and/or merging tasks, c) consider ranking and ordering items, etc. Thereafter, a survey developed by a Core Group (MSK, DB, LR and LH) with 19 open and closed questions (in 4 sections) was sent to all respondents. Section 4 included 4 statements about issues in bariatric exercise rated on a 0 (no importance) to 9 (imperative) scale. An example (the first statement) is, “Pre- and postoperative PA/exercise guidelines for bariatric surgery patients are needed”. Respondents were permitted another 2 weeks to respond. “Consensus” was defined by > 80% agreement for the first 3 sections of questions and inter-quartile range (≤ 2) for section 4. As part of this process, voting permitted the Core Group to later make minor clarifications to the model (Stage 6). A final model was presented to the entire panel, with an additional open comment period of 2 weeks ensuring representation of emerged categories.

In Stage 7, 4 authors (MSK, MD, OA, LR) established specific criteria to define each HOT [29], as seen in Supplement 5. These criteria defined what explicitly and implicitly constituted each higher-order category, and what explicitly was not part of each category. A new form/template was then created to re-screen the searchable pdf of all manuscripts for tasks and their properties (e.g., program delivery mode: in-person, telemedicine; format: 1-on-1, group) in each category. The re-review and recode using these forms were completed by 2 authors (MSK, OA). In some cases, emails were sent to the corresponding authors for clarification on study details.

## Results

A total of 63 studies were found describing exercise programs in MBS, representing 50 unique interventions (many studies had more than one published report). A further search to include all articles related to these interventions increased the total to 71 with 57% published after 2015. Over half of the studies were from just 2 countries – the USA (39%) and Brazil (14%). Exercise programs were mainly conducted in teaching hospitals (38%). The majority of articles were “proof of concept” (i.e., addressing the question of whether exercise is beneficial for weight loss) as opposed to implementation. The main component of these programs was supervised exercise training (65%). Later, article additions increased this total to 83 studies, which were further analyzed. See the Tables and Reference list in Supplement 6.

### Classification and qualifications of exercise professionals

The exercise professional delivering the PA programming was firstly an exercise physiologist in the USA (28%; person with a graduate degree in exercise physiology and/or certification) and a physiotherapist/physical therapist in the rest of the world (43%). A number of other exercise professionals (e.g., physical educators, coaches, personal trainers) were also involved. Qualifications and credentials were difficult to ascertain due to under-reporting. However, it was discovered (through details in studies and/or email inquiries) that 6 studies used bachelors-level exercise professionals, 9 used Masters-level, 9 used PhDs, and 2 used medical doctors (MD). In 7 cases, exercise physiologists were specifically credentialed by the American College of Sports Medicine (ACSM) and in one case by the National Strength and Conditioning Association (NSCA).

### Task data extraction

Data extraction resulted in 385 tasks across all studies, 43 of which were unique. The 5 most common tasks were as follows: real-time supervision of exercise (36 studies), fitness testing (31), exercise prescription (27), heart rate monitoring (21), and physical activity counseling (21). Less commonly described in the literature were exercise prescription for home, exercise behavioral counseling, and metabolic testing.

### Content analysis/card sort

Respondents sorted tasks into an average of 11.8 bins, each bin had an average of 3.6 items, and there was an average of 1.8 items deleted per respondent. Respondents suggested 34 items to add (3.4/respondent). Unique items were considered in later rounds. See Supplement 7.

A heat map of the associations of the original 43 exercise professional job tasks is provided in Supplement 8. The items with the strongest association (9 out of 10 raters agreed) were strength testing and physical function testing. Eight out of 10 raters indicated that the following 3 tasks were paired: strength testing/balance testing, fitness testing/function testing, prescription for home/prescription-individual. Seven raters agreed on 5 pairs, 6 raters - 10 pairs, 5 raters - 17 pairs, etc.

The hierarchical cluster analysis based on this matrix generated 2 models, which are shown in Supplement 9. Model B had a better fit index than Model A, as quantified by the cophenetic correlation coefficient (c = .85 and .50, respectively), a common statistic that measures the faithfulness with which the similarities between individual items in the original matrix were represented visually in the final dendrogram.

### Delphi process/Core Group

**Fig. 2.**
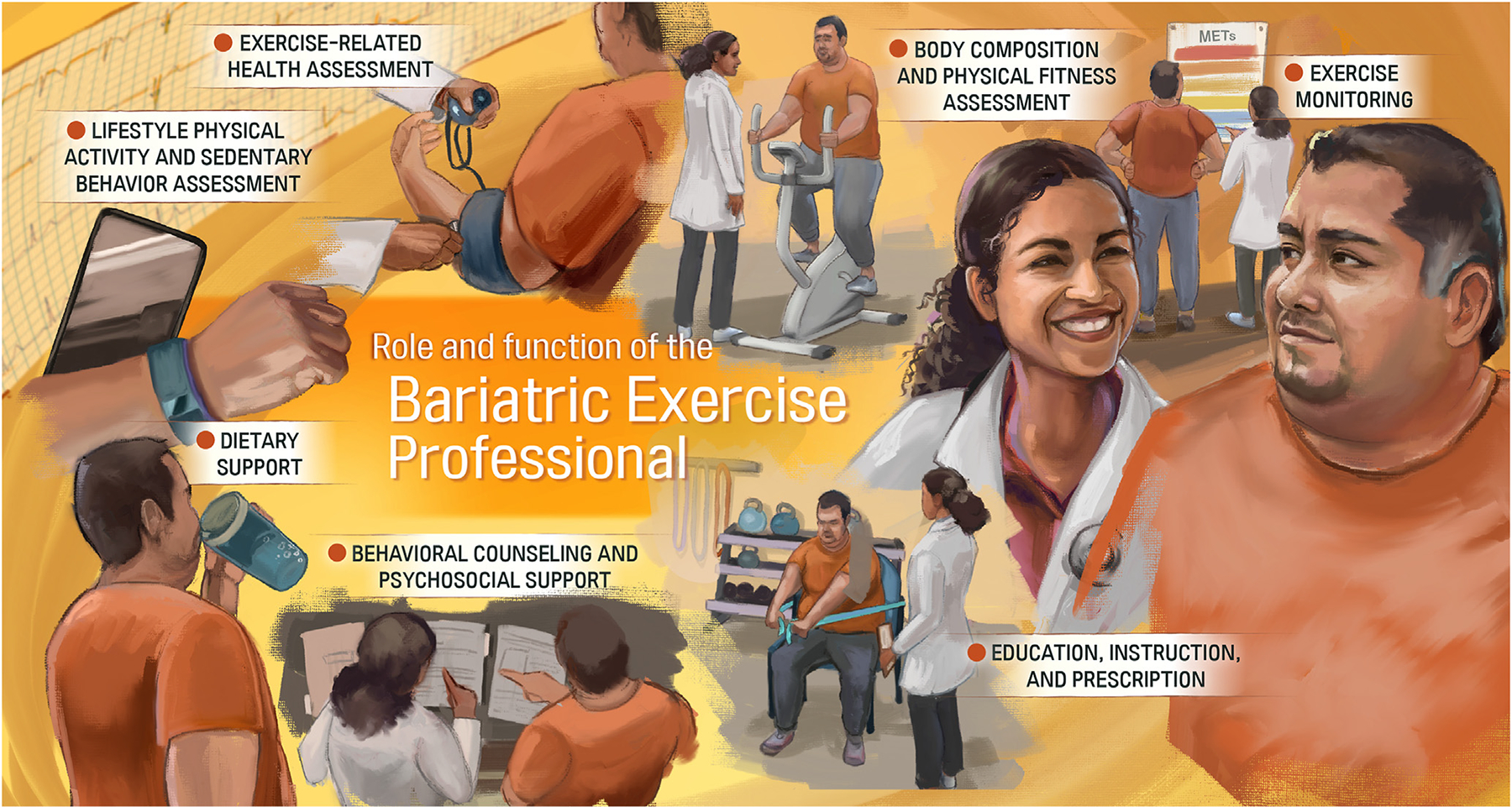
Illustrated final model (Version B) for clinical use. Certain aspects of tasks may be outside the scope of training for some specific exercise professions, and patients with multiple comorbidities would be better served seeing a clinical exercise physiologist as opposed to a personal trainer.

Respondents approved Model B. They indicated that “Passive stretching” should be eliminated and “exercise prescription adherence” should be included. The Core Group eliminated the general “prescription” task. Therefore, the final model included 42 patient-centered exercise tasks (i.e., lower order themes), which were divided into 14 higher order themes and 7 SHOTs. The final SHOTs were: (1) Exercise-related health assessment, (2) Body composition and physical fitness assessment, (3) Lifestyle physical activity and sedentary behavior assessment, (4) Education, instruction, and prescription, (5) Exercise monitoring, (6) Behavioral counseling and psychosocial support, and (7) Dietary support. The final model passed with a unanimous final vote. See Table 1, Figure 2, and Supplements 10–11 for the final model, with descriptions of the SHOTs. Task categories were not ranked, per group consensus, but were further sorted by temporal precedence in a clinical setting (starting with basic intake assessments).

### Survey: issues in bariatric exercise

The following statements were rated an average of 9.0, indicating “imperative” was rated by all respondents: 1) “Pre- and postoperative PA/exercise guidelines for metabolic and bariatric (MBS) patients are needed”, 2) “MBS programs need to include PA/exercise as part of multidisciplinary care”. The following questions were rated just by USA respondents, also receiving unanimous agreement that they are “imperative”: 1) MBS patients should receive customized and individualized PA/exercise guidance from credentialed exercise professionals with expertise in PA prescription and intervention, and 2) Exercise services in MBS settings should be standardized and reimbursed.

### Re-review

The higher-order task categories (HOTs) most represented in the literature were “Real-time monitoring, instruction and feedback” at 73%. The task HOT least represented was “stress testing” at 34%. In the review of differences in tasks performed by profession, 2 task HOTs were different. Exercise physiologists were determined to perform both Psychosocial support (HOT 13) and Dietary support (HOT 14) to a greater degree. See Supplement 12.

## Discussion

Increased PA, including exercise, is essential for the long-term success of patients undergoing MBS and helps to maintain health improvements even in the short-to-medium term [4–6,8,38]. However, many patients do not become active after surgery. Supports that could offset this inactivity (e.g., PA prescription, counseling, supervised exercise programs) are often not provided as part of clinical care. While there is a clear need for exercise professionals in the multi-disciplinary care of patients undergoing MBS, the specifics of their professional background and tasks they perform in a bariatric setting are unclear. To better clarify the roles and rationale for exercise professionals in MBS, we conducted a unique and rigorous multimethod analysis of all exercise programs peri-MBS. We extracted tasks of the exercise professional, submitted these to an expert group of clinicians for sorting into themes, used a sophisticated analysis to create a hierarchical model of these job tasks, and further consulted the expert group for their unique perspectives.

Overall, our study found exercise professionals perform a large set of job tasks related to the clinical care of the patient. Forty-three identified tasks were classified into 14 higher-order themes and then 7 super higher-order themes. These 7 included the following: (1) Exercise-related health assessment, (2) Body composition and physical fitness assessment, (3) Lifestyle physical activity and sedentary behavior assessment, (4) Education, instruction, and prescription, (5) Exercise monitoring, (6) Behavioral counseling and psychosocial support, and (7) Dietary support. The most common skills reported in the literature were: supervision of exercise, fitness testing, exercise prescription, heart rate monitoring, and physical activity counseling. However, the expert group decided to not rank tasks or their categories by commonality but treat them as relatively equal in importance to avoid overlooking other key functions of the exercise professional. Tasks conducted by exercise professionals in bariatric treatment align closely with similar practice in other clinical settings. For instance, our 7 SHOT categories overlapped considerably with core components categories from cardiac rehabilitation (e.g., Psychosocial Management, PA Counseling, Exercise Training Evaluation) [39,40] and with performance domains from the American College of Sports Medicine (ACSM) clinical exercise physiology credential (e.g., Exercise Testing, Exercise Prescription, Exercise Training and Leadership, Education and Behavior Change) [41]. The expert group noted that some tasks should not be included, such as postoperative PA clearance (frequently provided by an advanced practice registered nurse [APRN], physician’s assistant or surgeon). The group also indicated it might be worthwhile including other psychosocial support factors as well, including lessening stigmatization, which may contribute to PA/exercise avoidance in bariatric patients [42,43]. Overall, the results indicate that the exercise professional may have skills and abilities to provide a larger role than that suggested by Mechanick [44] and others [45], which appear to limit tasks to “lifestyle medicine evaluation”, physical activity counseling, monitoring adherence to PA guidelines, and supervising exercise sessions.

How exercise professionals are classified ostensibly varies by country. Research reviewed in this study demonstrated the exercise professional was most often an exercise physiologist in the USA, and a physiotherapist/physical therapist outside the USA, the 2 professions noted by the ASMBS Integrated Health Section [46]. It is possible that physical therapists are frequently utilized due to reimbursement issues, but we were unable to determine this in the current analysis. It is important to note that in Australia, Canada and the United Kingdom, like the USA, exercise physiologists work independent of physical therapists [47,48], and in some countries, like Singapore, they may work together. In Brazil, the exercise professional was typically a physical educator. Regardless, our analysis determined that the job tasks exercise professionals conducted in MBS differed on only 2 dimensions (i.e., Psychosocial support, Dietary support). The expert group did not make any specific recommendations regarding profession, credentialing, or qualifications, but it is clear that credentials of the exercise professional (e.g., exercise physiologist [also known as EP] versus clinical exercise physiologist [CEP]) should match the clinical complexity characteristics of the patients. Moreover, the experts rated as “imperative” that bariatric patients should have customized exercise guidance from credentialed exercise professionals (regardless of whether the professional is an exercise physiologist, physical therapist, or other qualified professional), and exercise services in the MBS setting should be incorporated into the reimbursable services of care.

The current study has several notable strengths, the first of which is the broad, robust methodological approach used to address the topic, which provided several levels of confirmative evidence for the results. The process was inductive, starting with the literature, and the tasks identified were categorized and refined by an expert group that included a multidisciplinary team of exercise practitioners, scientists, nurses, surgeons, and dietitians from multiple nations. Moreover, the bariatric exercise practitioners involved have several decades of combined experience in this area.

This investigation had some limitations. Studies in the review were predominantly research- and not clinical-focused. As a consequence, most of the patients in these articles were higher functioning – excluding individuals with mobility problems, heart issues, and mental health problems and thus limiting the generalizability of the findings. Thus, one might presume that the literature does not describe a true clinical sample, which may have had implications for the tasks of the exercise professional. To check our model of tasks, a full-scale survey of exercise professionals in MBS across the world should be done as a next step. While highly qualified and experienced, the expert group was a convenience sample, with the implication that there was likely some bias in the analysis. Lastly, a majority of studies provided insufficient reporting of exercise-related tasks. The notable exceptions of this were from a group in Spain [28,49] and the USA [50]. However, we aimed to compensate for this by including as many studies as possible on the topic. Furthermore, we contacted numerous authors of the included publications, and their responses are included in these data. Despite these great efforts, it is possible that we missed some important tasks, such as program development or promotion of a physically active environment.

The results of this study have important clinical implications. First, these models will provide a clearer understanding of the role of the exercise professional in the MBS setting and the value they bring to a multidisciplinary surgery team [46,51,52]. More practically, findings provide practical and useful information that could contribute in 4 major areas: 1) describing the roles and responsibilities exercise professionals are able to fill, helping create job descriptions, 2) developing CPT/billing codes for reimbursement (aligning with how cardiac rehab was developed), helping to spur other funding for positions, 3) credentialing exercise professionals in bariatric care and development of “core competencies,” [40] and 4) helping to coordinate care from clinical exercise personnel, perhaps in a system of coordinated exercise care. Future needs include PA guidelines for patients undergoing MBS, perhaps similar to those from Exercise and Sports Science Australia [3] or those recently established for type 2 diabetes [53]. To this end, it is important to generate more experimental data from well-designed studies on the greater role of PA/exercise in the maintenance of body mass after surgery, as well as other outcomes, and to clarify the ideal type, duration, intensity, and progression of such exercise. There also needs to be better reporting of roles and tasks performed by the exercise professional in surgery, as outlined by the CERT guidelines [28,49].

Future work should also describe the following: 1) important knowledge, skills, and competencies (e.g., identifying appropriate exercise protocols based on various health conditions and physical limitations, monitoring blood pressure during exercise and testing, resting and exercise ECG, etc.) [54], 2) how tasks vary with surgical benchmarks (i.e., pre, post and long-term after surgery) and types of programming (e.g., supervised exercise training versus PA counseling, 3) scope of practice, and 4) how exercise professionals collaborate as a member of the multidisciplinary team commonly comprised of nurses, dietitians, psychologists, and other providers. For instance, it is likely the case that some tasks described in our model are not solely the responsibility of the exercise professional. More studies are needed to describe the translation and implementation of bariatric and medical weight management PA programs for standardization, reproducibility and to strengthen the case for the role of the exercise professional in MBS and even the greater realm of healthcare.

## Conclusion

To our knowledge, this is the first study to systematically investigate the role of the exercise professional in a metabolic and bariatric surgery (MBS) setting. Exercise is regarded as an important facet of multidisciplinary surgical care, but has been poorly implemented, and many programs do not have an exercise professional within their team. It is also important to note that patients want access to exercise professionals before and after their surgery [55], which, altogether underpins why we completed the first systematic and multimethod investigation of the exercise professional in MBS. We searched for and analyzed every published bariatric exercise/PA program from inception to 2022. Our expert group of bariatric exercise professionals, allied clinicians, and scientists reached a consensus on 7 major classifications of patient-centric job tasks for the exercise professional. Tasks varied marginally by the specific field of the exercise professional (e.g., exercise physiologist, physiotherapist/physical therapist, others). Results from our study provide convergent validity with other exercise professional credentialing bodies. We believe that data from this rigorous and unique analysis helps to fill an important gap, enabling continued progress to solidify these positions, while informing new and more precise recommendations on supporting and standardizing comprehensive exercise treatment for obesity. It is also important for governing medical associations across the world to formally recognize experienced exercise professionals as playing pivotal roles in the overall multidisciplinary healthcare for patients undergoing MBS surgery.

## Supplementary Material

Supplement 3

Supplement 1

Supplement 2

Supplement 5

Supplement

Supplement 6

Supplement 7

Supplement 8

Supplement 9

Supplement 10

Supplement 12

Supplement 11

Supplement 13

Supplement 14

## Figures and Tables

**Table 1:** Final model with descriptions of the Super Higher-order Task (SHOT) themes

Super Higher-order task theme (SHOT) #	Super Higher-order task theme (SHOT)	SHOT Description/Interpretation	Higher-order task theme (HOT) #	Higher-order task theme (HOT)	Lower-order Task Theme (LOT) #	Lower-order Task Theme (LOT)

1	Exercise-related health assessment	Perform pre-participation screening, and identify comorbidities. Conduct baseline assessments of vitals. Utilize established and valid protocols to determine aerobic fitness and cardiac risk. Interpret data and track progress.	1	Risk factor and needs identification	1	Risk factor identification
					2	Needs assessment
			2	Exercise-related health assessment	3	Stress testing
					4	Vitals
					5	ECG & other cardiopulmonary
2	Body composition and physical fitness assessment	Assess fat and lean mass. Evaluate muscular strength, endurance & power, balance, and other indicators of physical fitness and function. Interpret data to utilize for prescription and as indicators of program effectiveness.	3	Body composition assessment	6	Body composition testing
					7	Anthropometry
			4	Fitness testing	8	Fitness testing
					9	Balance testing
					10	Strength testing
					11	Physical function testing
3	Lifestyle physical activity and sedentary behavior assessment	Assess 24-hr, free-living physical activity behavior, including sedentary behavior, light, moderate and vigorous activity. Evaluate for baseline and tracking over time with validated surveys, wearables and/or accelerometry. Measure steps, sitting time and caloric expenditure. Interpret data to utilize for prescription and as indicators of program effectiveness.	5	Lifestyle physical activity and	12	PA/Sedentary assessment
				sedentary behavior	13	PA/Sedentary monitoring
				assessment (same as SHOT)	14	Accelerometry
4	Education, instruction, and prescription	Promote literacy of fitness and its effect on health. Educate patients in principles of enhancing physical activity and reducing (and breaking up) sedentary time across the 24-hr day. Guide patients in exercise basics (e.g., specificity, overload) to promote fitness and health outcomes in the clinic and independently at home (selfmanagement). Conduct safe, adaptive and individualized programming to ensure continued progress.	6	Fitness and health education	15	Fitness and Health Education
			7	Physical activity instruction	16	Orientation
					17	Exercise training
					18	Exercise instruction
			8	Exercise prescription	19	Prescription-individual (in clinic)
					20	Prescription-home (free living)
					21	Prescription-group (in clinic)
			9	Personalization	22	Modification
					23	Individualization
					24	Progression
					25	Adjustments
5	Exercise monitoring	Supervise group and/or individual exercise training. Monitor hemodynamic and metabolic responses, signs and symptoms with both subjective/self-report and objective (both active and passive) instruments, including wearables, continuous glucose monitoring (CGM) and other digital assessments.	10	Metabolic assessment	26	Metabolic testing
					27	Glucose testing (clinic/lab)
					28	Glucose monitoring (peri-exercise)
			11	Monitoring	29	Symptom monitoring
					30	HR monitoring
					31	Supervision (group)
					32	Monitoring (individual)
					33	Exercise monitoring
					34	Effort/Exertion monitoring
6	Behavioral counseling and psychosocial support	Promote physical activity with basics of behavior change, such as goal setting, social support, and reinforcement, within the scope of profession. Demonstrate encouragement and follow up for continued success. Reinforce and support psychosocial recommendations from licensed psychology and social work professionals. Refer to these professionals when indicated/necessary.	12	Counseling techniques	35	PA/sedentary counseling
					36	Motivation & feedback
					37	Goal setting
					38	Exercise prescription adherence
			13	Psychosocial support	39	Support group
					40	Psychosocial assessment
7	Dietary support	Reinforce and support dietary recommendations from licensed nutrition professionals. Support nutrition basics, such as energy balance and exercise-related nutrient monitoring, within the scope of profession. Refer to nutrition professionals when indicated/necessary.	14	Dietary support (same as SHOT)	41	Food monitoring
					42	Food assessment

## Data Availability

All data produced in the present study are available upon reasonable request to the authors.
