# Supplementary material for: Role of the exercise professional in metabolic and bariatric surgery
Source: Surg Obes Relat Dis. Author manuscript; Available in PMC 2025 Jan 1. (PMC11311246; doi:10.1016/j.soard.2023.09.026)
Supplement: Supplement 3 [file NIHMS2008743-supplement-Supplement_3.pdf]

Supplement 3. Data extraction protocol

Two investigators (MSK and MD) performed the data extraction (Stage 2), which first proceeded for information related to the "who" of the exercise program (i.e., type of exercise professional who delivered physical activity programming). Any information regarding education, clinical training, credentialing, and affiliation of the exercise professional was also noted. Gathering this type of data presents some difficulty, because: a. most research articles do not directly state what provider/person completed each aspect of the study, b. authorship lists on most studies represent individuals from diverse academic backgrounds, c. not all study personnel are acknowledged, d. the term used to describe exercise professionals varies across geographical regions, such as: exercise physiologist, physical therapist, exercise specialist, trainer, health educator, etc., and e. missing information due to journal space constraints. Consequently, for studies going back to 2010, the corresponding author in each study's byline was emailed by the first author with follow up questions.

These same investigators then extracted data related to patient-centered (clinical) exercise and physical activity program tasks completed by the exercise professional. To accomplish this, we decided to use a more inductive approach – relying on the bariatric exercise literature itself and not depending, a priori, on definitions of job tasks as stated by documents from various professional organizations about the role of exercise practitioners (1-4), which may or may not be applicable, or may be inaccurate or insufficient in this setting. By "clinical" or "patient-centered", it is meant that we did not attempt to focus on tasks related to basic science questions (e.g., laboratory assays), administration, or other activities not directly related to patient contact.

An initial list of job tasks was identified through three means. First, by identifying instances where the role of the exercise professional were directly stated (usually as preceded by strong action verbs) (i.e., created the exercise program, screened patients, completed assessments, supervised/monitored training, provided counseling). Second, in studies conducted entirely by exercise physiologists, one would presume that all activities were conducted by these exercise professionals. Third, a corresponding author declared such actions in a personal communication. Two investigators systematically evaluated and interpreted publications independently to minimize bias (MSK and MD).

References

1. American Physical Therapy Association. About Physical Therapists and Physical Therapist Assistants 2023 [Available from: https://www.choosept.com/why-physical-therapy/about-physical-therapists-and-physical-therapist-assistants.
2. American College of Sports Medicine. ACSM Certified Exercise Physiologist: Exam Content Outline 2023 [Available from: https://www.acsm.org/docs/default-source/certification-documents/acsmep_examcontentoutline_2020.pdf?sfvrsn=d413f75_16.
3. Froment FP, Olson KA, Hooper TL, Shaffer SM, Sizer PS, Woodhouse LJ, et al. Large variability found in musculoskeletal physiotherapy scope of practice throughout WCPT and IFOMPT affiliated countries: An international survey. Musculoskeletal Science and Practice. 2019;42:104-19. doi: https://doi.org/10.1016/j.msksp.2019.04.012
4. World Confederation for Physical Therapy. Description of physical therapy: Policy statement. 2019.
