# Supplementary figures and images for: Role of the exercise professional in metabolic and bariatric surgery
Source: Surg Obes Relat Dis. Author manuscript; Available in PMC 2025 Jan 1. (PMC11311246; doi:10.1016/j.soard.2023.09.026)

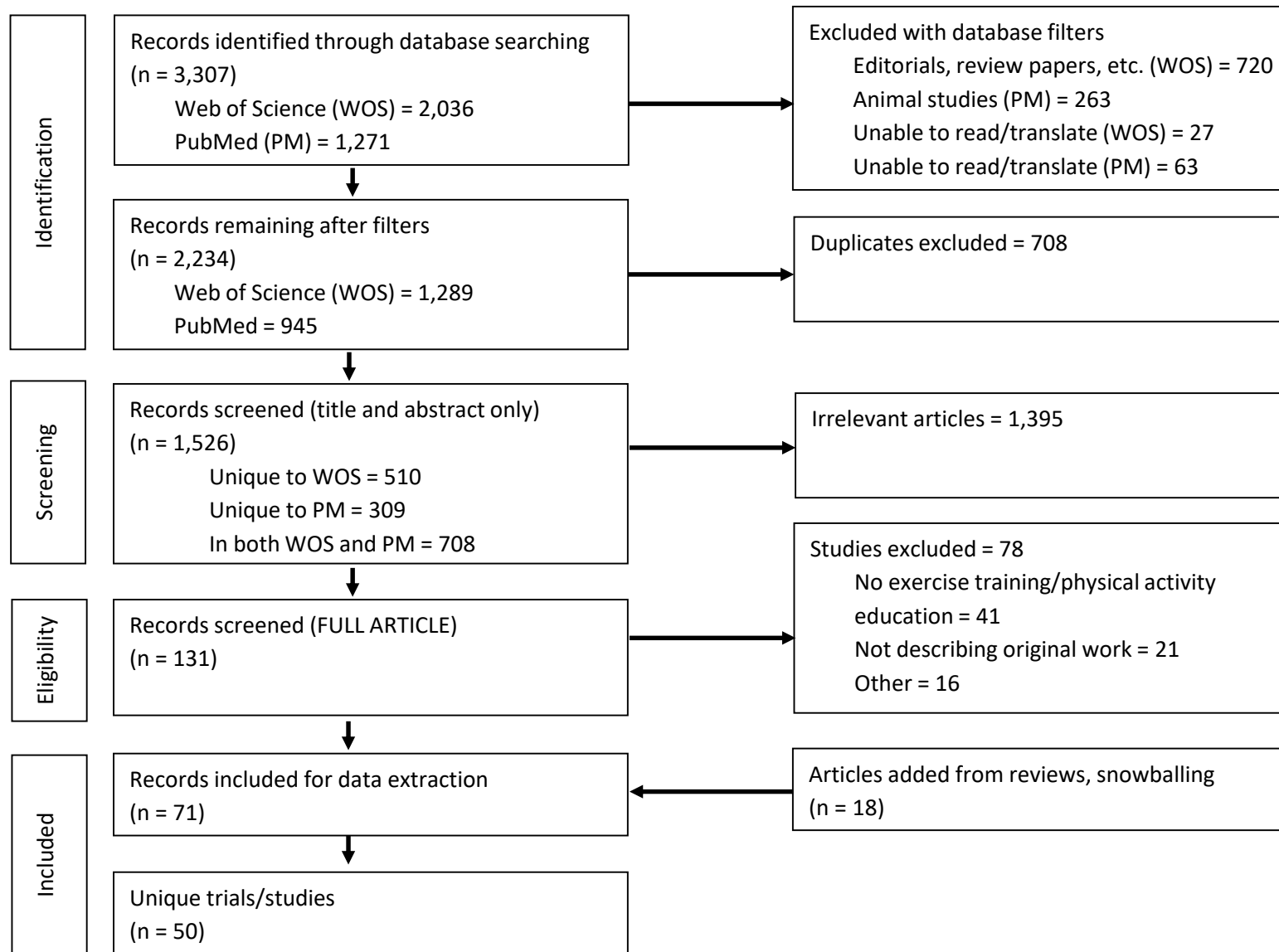

Supplement 1. Prisma CONSORT diagram demonstrating the literature search.

Supplement: Supplement 1 [file NIHMS2008743-supplement-Supplement_1.pdf]

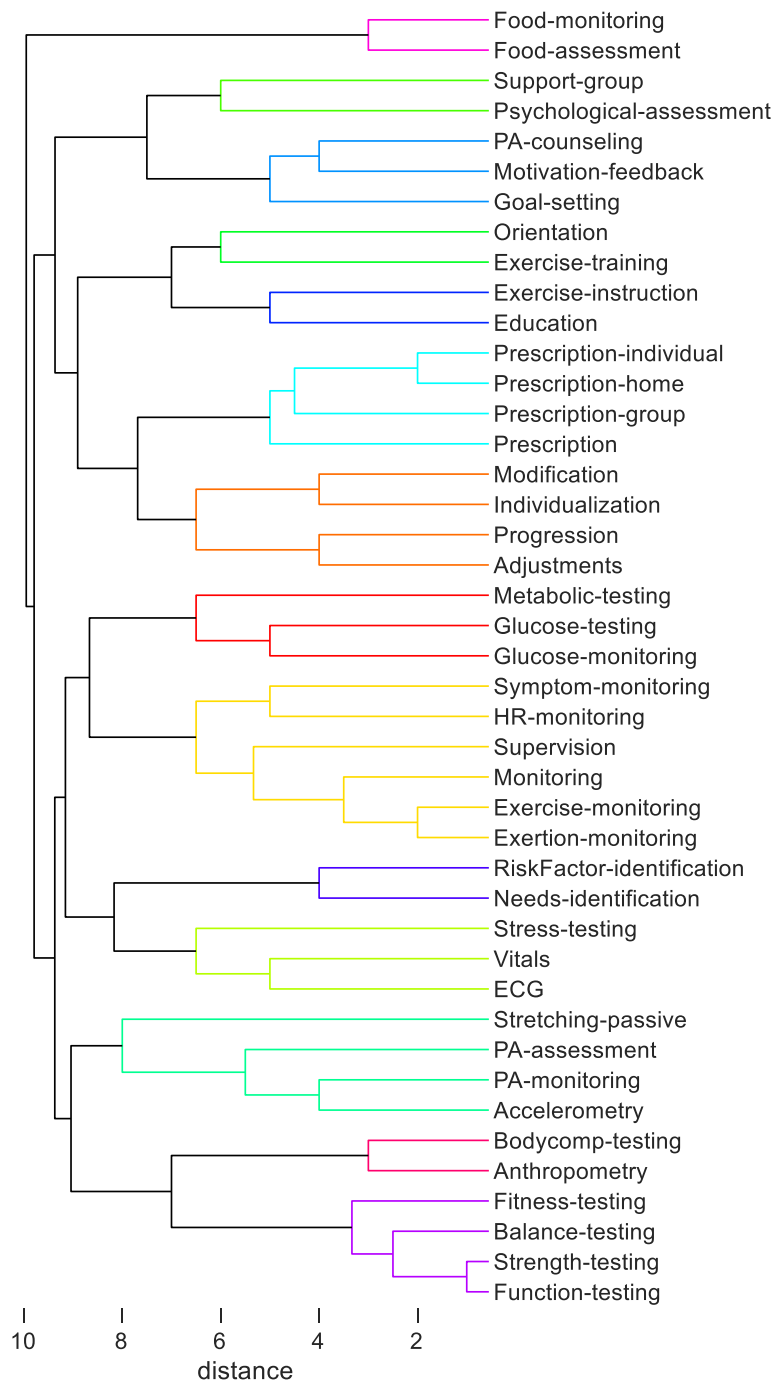

Supplement 10. Final hierarchical cluster analysis model approved in the Delphi process

Supplement: Supplement 10 [file NIHMS2008743-supplement-Supplement_10.pdf]

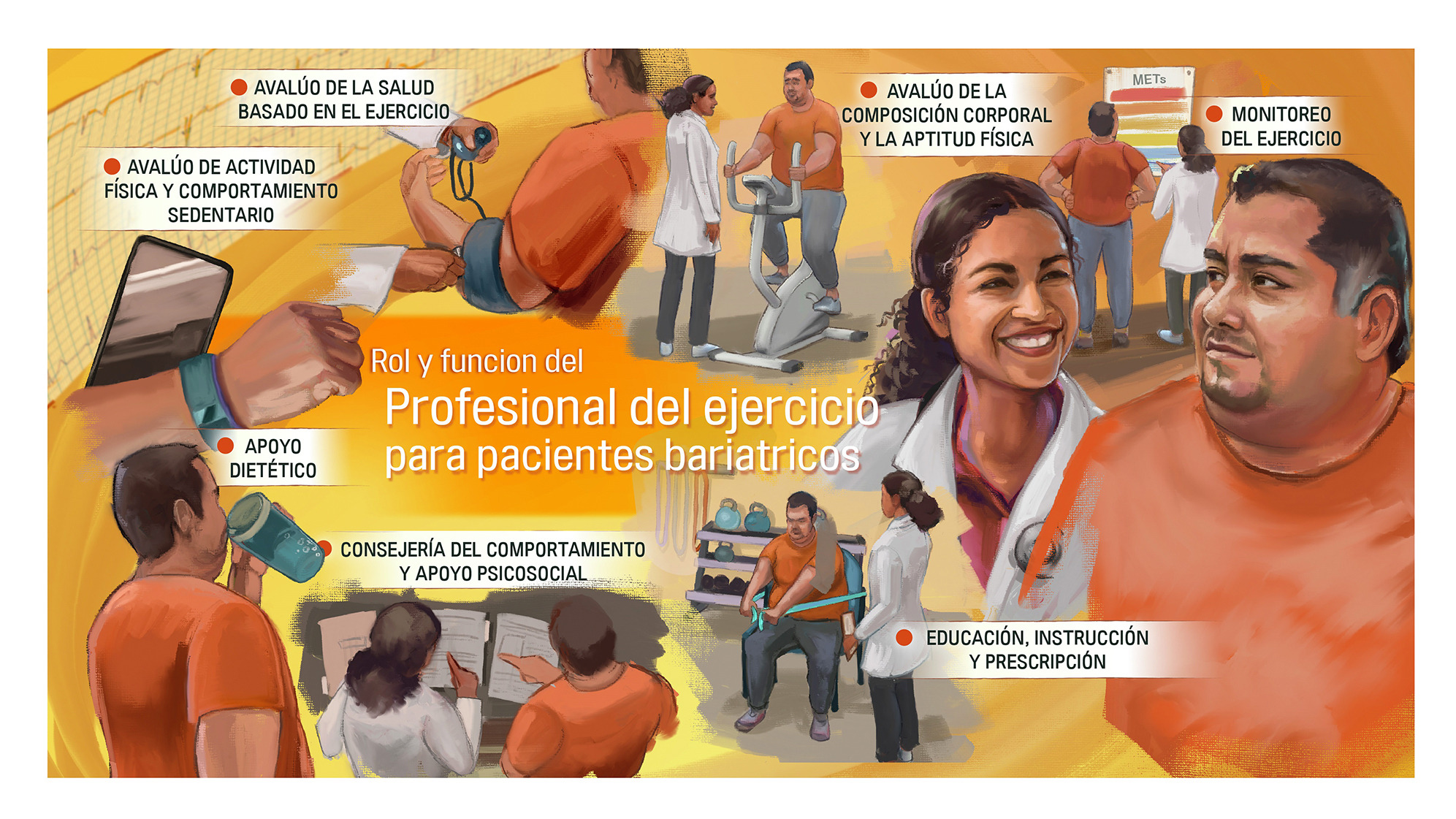

Supplement: Supplement 13 [file NIHMS2008743-supplement-Supplement_13.jpg]

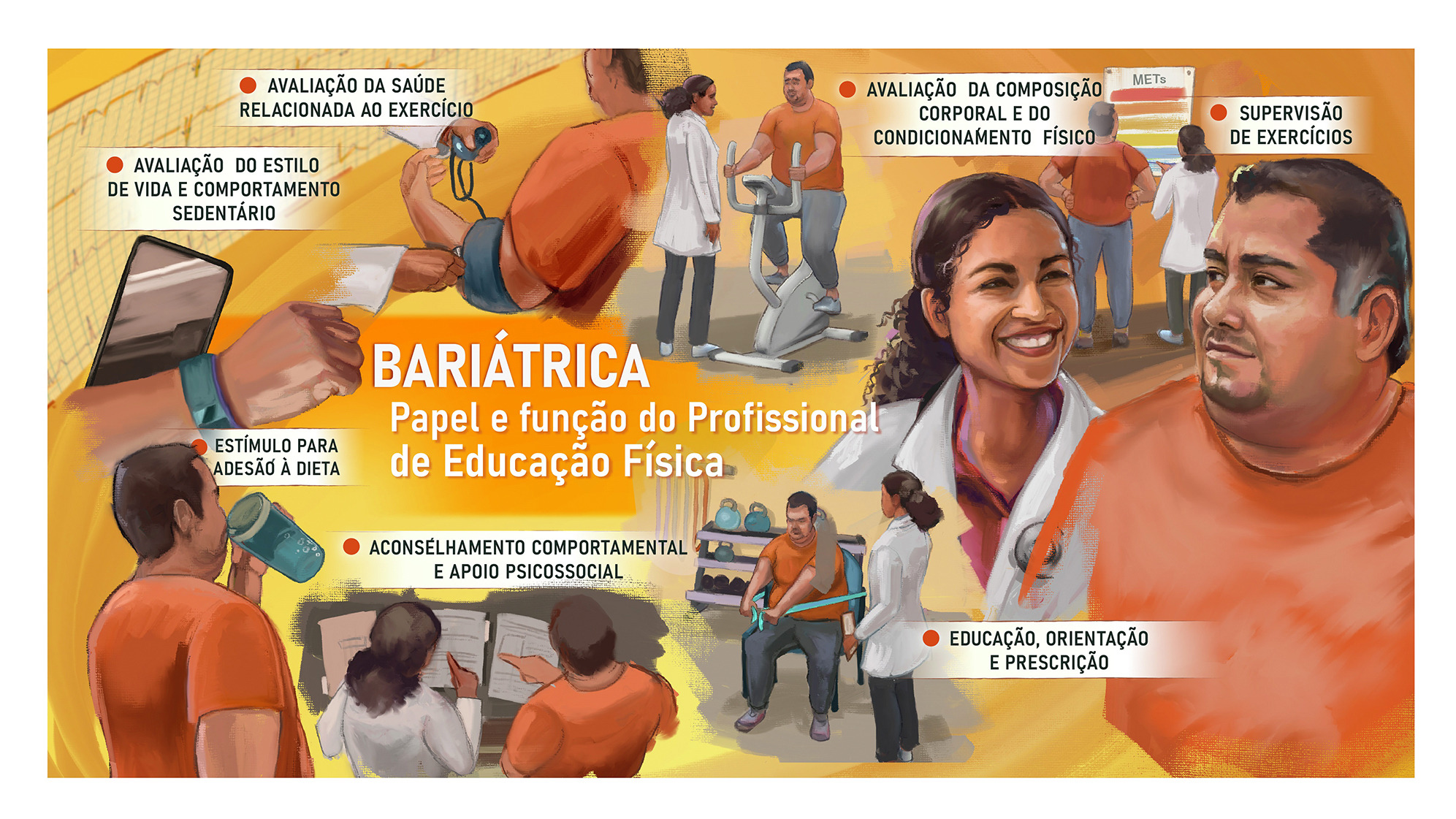

Supplement: Supplement 14 [file NIHMS2008743-supplement-Supplement_14.jpg]
