# Supplementary material for: Role of the exercise professional in metabolic and bariatric surgery
Source: Surg Obes Relat Dis. Author manuscript; Available in PMC 2025 Jan 1. (PMC11311246; doi:10.1016/j.soard.2023.09.026)
Supplement: Supplement 5 [file NIHMS2008743-supplement-Supplement_5.pdf]

Supplement 5. Criteria for task identification and classification.

	Task Category from Table 2	Does NOT include	Explicit (X symbol)	Implicit (/ symbol) suggesting strongly that it was conducted	Notes and search criteria
1	Risk Factor and needs identification	Does **not** include:<br><br>Limitations due to age, weight status and various eligibility factors.<br><br>Signed approval from PCP/surgeon for exercise	1. Exercise health & musculoskeletal screening,<br>2. determination of contraindications<br>3. Needs identification<br>Includes:<br>PAR-Q,	Inclusion/ exclusion criteria suggestive that a clinical screening was conducted.	Search for words: RISK / SCREENING / NEEDS / CONTRAINDICATIONS<br><br>In some studies, no additional exercise-related health screening was conducted (e.g., Parikh; Rothwell)
2	Exercise-related health assessment	Does **not** include:<br>Functional testing, like 6-minute walk tests	physical by a sports physician<br>Vitals (Blood pressure) – e.g., Funderburk<br>Bruce protocols<br>Modified-Bruce protocols	VO2max data or similar was reported	
3	Body Composition Assessment	Does **not** include:<br>Does not include simple weight, height and BMI.	Tests of percent body fat and resting metabolic rate and waist and hip circumferences.	Data suggestive of test completion	
4	Fitness testing	Does **not** include:<br>"Perceived" fitness	Other non-graded exercise tests, like the 6MWT or the Cooper Test.	Data suggestive of test completion	
5	Lifestyle physical activity and sedentary behaviors assessment	Does **not** include:<br>Gym attendance	Accelerometry (i.e., Actigrpah);<br>Fitness trackers (i.e., Fitbits)<br>Pedometry<br>Diaries / logs of activity	Step data included	Search for words: STEPS
6	Fitness and Health Education	Does **not** include:<br>Information about the program	Structured exercise curriculum<br>Includes "exercise guidelines" as provided in a control group for a RCT.<br>Exercise instructions for home.	Evidence of curriculum (e.g., table of contents)	
7	Physical Activity Instruction	Does **not** include:<br>Structured exercise program	Includes:<br>Familiarization	List of exercises that would infer that patient would need instruction	

			Dumbbells, resistance bands, bands,		
8	Exercise Prescription	Does **not** include:<br><br>Outside physical activity that a patient would routinely conduct on their own.<br>Exercise counseling about exercise<br>Exercise or PA education	Any exercise outside of the structured/supervised training program, as provided by the exercise professional.<br>Includes access to facility during non-training times	Text highly suggestive that a home program was being conducted	Search for words: HOME, OUTSIDE
			Prescribed exercise training program defined by exercise variables, such as:<br>Minutes/time (duration)<br>Frequency of exercise<br>Type/Mode<br>Etc.<br>Early ambulation program	Text highly suggestive that a standardized program was being conducted.	
9	Personalization	Does **not** include:<br>General guidelines are given to all/most patients<br><br>Different guidelines/instructions for two or more different groups	Customization, individualization and/or modification based on needs/ health status<br>Modifications based on pain and tolerance<br>Implied if they use heart rate % to prescribe the exercise.<br>"Optimization" of exercise program	Heart rate zone provision<br><br>If a case study, it is assumed to be individualized for the patient.	Search for words:<br><br>Individualization, Modification, Customization
		Does **not** include:<br>Standardized exercise prescription that does not change over time.	Weekly, bi-weekly or monthly changes based on a variety of factors, like improvements in fitness or less limitations.	Adjustments made but the criteria by which the adjustments are made is not clear, or it is not applicable for all patients (only some were progressed).	Search for words:<br>Progression<br>Increment*
10	Metabolic Assessment	Does **not** include:<br>HR prescription only	Also includes BP and SPO2 and glucose<br>Additional action if parameters are out-of-expected range.	Data suggestive	Search for words:<br>Heart rate<br>Glucose<br>Blood sugar
11	Monitoring	Does **not** include:<br>Any activities in which patients were not within sight.	Includes orientation and familiarization and training to ensure safety and low abdominal pressure<br>Supervision / Supervised exercise training, including instruction, guiding and feedback<br>Teaching the Borg exertion scale to self-monitor	Text highly suggestive that feedback was being provided.	In many studies, it is not clear if some monitoring was passive or strictly active (i.e., passive monitoring from a desk/station at a gym vs. continuous supervision of each patient.

12	Counseling Technique	Does **not** include:<br>Education<br>Passing out guidelines<br>General advice	Instructions on goal setting, motivational interviewing or other techniques<br>Contingency planning ("if this happens, do this…")<br>Strategies to build confidence through experiences	Evidence of strategies (e.g., from a table)	
13	Psychosocial Support	Does **not** include:<br>Therapy<br>Other services necessitating psychiatry licenses	Onward Referral<br>Support of Psycho/Social Worker	Tables with psychological data in it in methods or results, information in regards to psychosocial assessments	
14	Dietary Support	Does **not** include:<br>Therapy<br>Other treatments necessitating licensing of dieticians	Education of basic consulting on bariatric dietary basics per-post surgery, (PPO(?) Supplements, Macros, Importance of vitamins); energy balance<br>Assessment<br>Onward referral	Lists of information about macronutrients, etc.	
