# Supplementary material for: Role of the exercise professional in metabolic and bariatric surgery
Source: Surg Obes Relat Dis. Author manuscript; Available in PMC 2025 Jan 1. (PMC11311246; doi:10.1016/j.soard.2023.09.026)
Supplement: Supplement [file NIHMS2008743-supplement-Supplement.pdf]

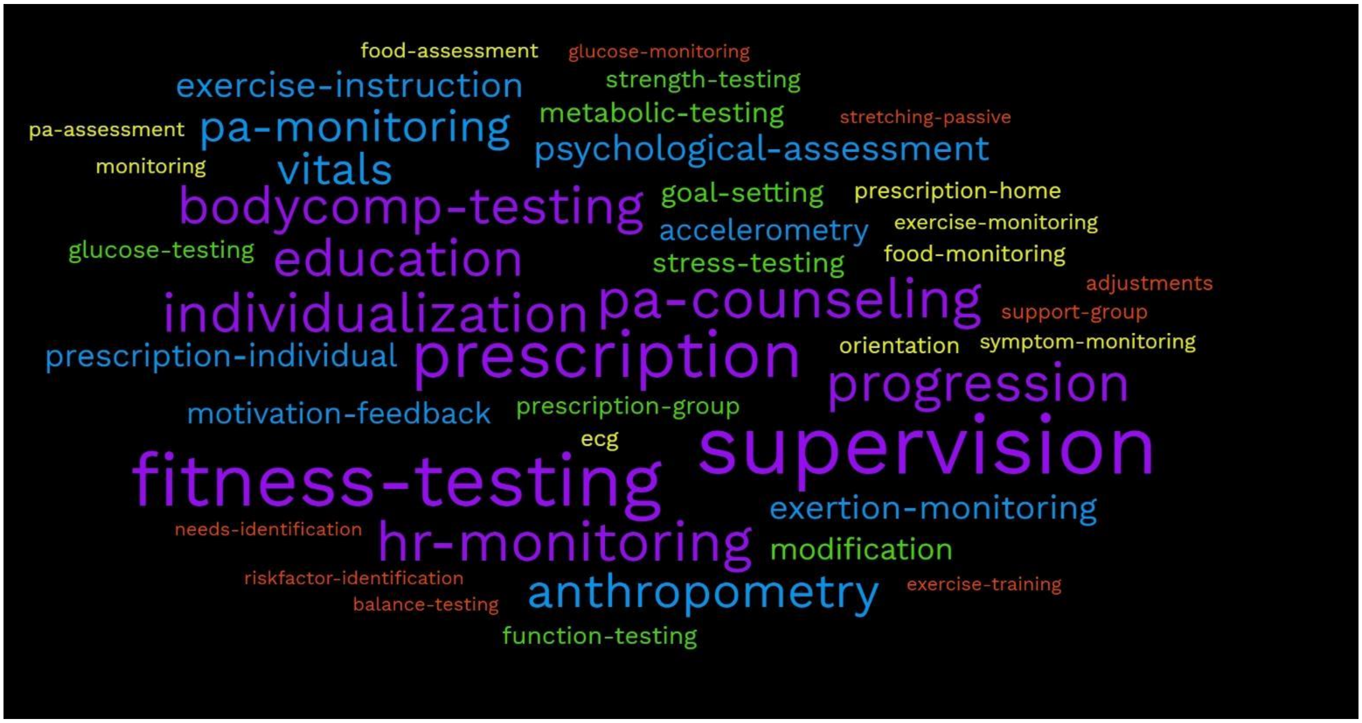

Supplement 4. Word cloud illustrating common exercise job tasks presented to the research team for the bin sort assignment.
