# Supplementary material for: Role of the exercise professional in metabolic and bariatric surgery
Source: Surg Obes Relat Dis. Author manuscript; Available in PMC 2025 Jan 1. (PMC11311246; doi:10.1016/j.soard.2023.09.026)
Supplement: Supplement 6 [file NIHMS2008743-supplement-Supplement_6.pdf]

Supplement 6 with citations (see below)

Table 1A. Details of studies from the USA

Studies # *	First author	Year	Included in data extraction (co-authors MSK and MD)	Study Type	Study Name	Country of corresponding author	Setting	Program Type	Program length	Mode of exercise training	Patient contact frequency	Exercise professional	Training/ education/ credentialing (if noted)
1	Berggren	2008	X	Prospective intervention	None	USA	University laboratory	Supervised exercise training	10 consecutive days	Endurance training	Daily	Masters-level graduate student	American College of Sports Medicine (ACSM)
2-4	Bond;<br>Bond;<br>Bond	2015 a;<br>2015 b;<br>2017	X	RCT	Bari-Active	USA	University hospital, at home	Behavioral intervention,<br>counseling, physical activity tracking	6 weeks	Home-based walking	1x/ week (weekly)	Doctoral-level graduate student	Master's in physical activity-related studies
5	Brandenberg	2005	X	Retrospective analysis	None	USA	University hospital	Behavioral intervention, supervised liquid supplement diet	6 weeks	Behavior modification component (non-exercise)	1x/ week (weekly)	Exercise specialist; psychologist	Not noted
6-10	Carnero;<br>Coen;	2017;<br>2015 a;	X	RCT	None	USA	Public hospital; university	Semi-supervised exercise training	26 weeks	Aerobic exercise (stationary	3-5x/ week with at least one directly supervised	Masters-level exercise physiologist	Master's in exercise physiol

	Coen;<br>Lopez;<br>Woodlief	2015 b;<br>2017;<br>2015								cycling or walking)	session per week		ogy (EP)
11	Coleman	2016		RCT	None	USA	Private hospital, at home	Supervised exercise training, phone counseling, daily pedometer, reading program curriculum, recording daily physical activities	26 weeks	Resistance training, aerobic exercise, flexibility training	2x / week with 3x/ week of self-directed exercise	Not specified	Not specified
12	Creel	2016	X	RCT	None	USA	Clinic	Exercise counseling with a pedometer	26 weeks	Walking	3x/ week (2 weekdays and 1 weekend day)	Not specified	Not specified
13	Daniels	2018	X	RCT	None	USA	University laboratory	Supervised exercise training	12 weeks	Resistance training	3x/ week	Doctoral-level graduate student	ACSM EP and Certified Personal Trainer (CPT), NSCA Certified Strength and Conditioning Specialist (CSCS)
14	Funderburk	2010	X	Quasi-experimental	None	USA	University hospital	Supervised exercise training	12 weeks	Aquatic exercise	2x/ week	Recreational therapist	Licensed and nationally certified

15	Gilberts on	2020		Pilot study	None	USA	At home	Unsupervised exercise training with exercise diary and activity tracker	30 days	Aerobic exercise (walking)	5x/ week	Not specified	Not specifi ed
16-18	Gill;<br><br>Powell;<br><br>Powell	2018;<br><br>2018;<br><br>2020	X	Qualit ative; prospe ctive interve ntion	BELT progra m; FLOW	USA	University, hospital	Supervised exercise training; behavioral change intervention	12 weeks; 16 weeks	Aerobic exercise, resistance training	3x/ week	Doctoral-level, registered clinical exercise physiologist (RCEP), Doctoral-level graduate students; assistant professor	ACSM , NASM
19-21	Halperin ;<br><br>Panosia n;<br><br>Hamdy	2014;<br><br>2017;<br><br>2018	X	RCT; review	Why WAIT; SLIMM-T2D	USA	Clinic; university hospital	Supervised exercise training; unsupervised exercise training	12 weeks	Aerobic exercise, resistance training, flexibility training	3-4x/ week (Weeks 1-4); 5x/ week (Weeks 5-8); 6x/ week (Weeks 9-12); Weekly	RCEP	Master s in kinesio logy and nutritio n
22	Hickey	1999	X	Prosp ective interve ntion	None	USA	University laboratory	Supervised exercise training	7 days	Aerobic exercise (treadmill and cycle ergometer)	Daily	Masters-level graduate student	ACSM
23	Huck	2015	X	Quasi-experi mental	None	USA	University laboratory, private community-based training facility	Supervised exercise training	12 weeks	Aerobic exercise, resistance training, stretching	2x/ week (Weeks 1-6); 3x/ per week (Weeks 6-12)	Graduate professor; CSCS, CPT	PhD

24	Kalarchian	2013	X	RCT	None	USA	University hospital, bariatric clinic	Physician-supervised diet and exercise training	26 weeks	Participants were given a goal of 30 minutes of physical activity (PA) at least 5 days per week (non-exercise)	12 individual, face-to-face sessions and 12 telephone contacts	Non-exercise professionals	Not specified
25	Kelley	2006	X	Editorial	Exercise Right	USA	Hospital	Unsupervised exercise training	6 weeks	Not specified	Not specified other than post evaluation and quarterly or semiannual basis follow-up assessments	Exercise physiologist	Bachelor's degree
26	Kerrigan	2012	X	Case study	None	USA	Hospital, bariatric center	Unsupervised exercise training	8 weeks	Aerobic exercise (walking, treadmill, recumbent stepper, swimming), chair aerobics	3-4x/ week	Clinical exercise physiologist	Not specified
27	Klasnja	2020	X	Pilot study	BariFit	USA	Public hospital	Mobile health intervention	16 weeks	Adaptive step goals	Daily	Research specialist	Not specified
28	Parikh	2012	X	RCT	None	USA	University hospital	Medically supervised weight management, nutrition, physical activity education,<br><br>individualized behavior modification counseling	26 weeks	Not specified	Monthly	Dietitian	Not specified
39	Shah	2011	X	RCT	None	USA	University hospital fitness center	Semi-supervised exercise training with exercise diaries and heart rate monitors	12 weeks	Aerobic exercise	5x/ week (with 1-2 days at the fitness center)	Exercise physiologist	PhD

30	Zagarins	2011	X	Descri ptive	None	USA	Hospital, at home	Supervised exercise training	12 weeks	Aerobic exercise (walking, treadmills, recumbent exercise bicycles, ellipticals), resistance training	1x/ week (weekly)	Exercise physiologist ; CPT	Not specifi ed

* Various trials/studies resulted in multiple publications, which were combined.

** National Strength and Conditioning Association (NSCA), Certified Strength and Conditioning Specialist (CSCS)

Table 1B. Details of studies from outside the USA

Studies # *	First author	Year	Included in data extraction (co-authors MSK and MD)	Study Type	Study Name	Country of corresponding author	Setting	Program type	Program length	Mode of exercise training	Patient contact frequency	Exercise professional	Training/ education/ credentialing (if noted)
1-4	Auclair; Lemiux-Simard; Pettigrew; Tardif	2020; 2021; 2019; 2020	Auclair – X<br>LS – NO<br>Pettigrew - NO<br>Tardif - X	RCT	ACTIVE	Canada	University hospital	Supervised exercise training	12 weeks	Aerobic exercise, strength training	3x/ week	Clinical Exercise Specialist (CES)	ACSM
5-8	Baillot;<br>Baillot;<br>Baillot;<br>Baillot	2013;<br>2016;<br>2016;<br>2018	X	Pilot study; RCT	PreSET; TelePreSET	Canada	Hospital; at home	In-person and virtual semi-supervised exercise training	12 weeks	Endurance training, strength training, Aquagym	2-3x /week on site (+ sessions at home if missed)	Doctoral-level kinesiologist	Not specified
9	Brown	2016	X	Prospective intervention	None	Australia	University hospital	Virtual behavioral education	1 year	Not specified	3x/ week	Nurse manager	Not specified
10	Brun	2019		Longitudinal study	None	France	University hospital	Supervised exercise training	5 years	Endurance training	3x/ week	Not specified	Not specified
11	Campanha-Versiani	2017	X	Quasi-experimental	None	Brazil	University hospital	Supervised exercise training and unsupervised training	36 weeks; 1 year	Weight-bearing exercise, aerobic exercise	2x / week	Not specified	Not specified

12-13	Castello;<br><br>Castello-Simões	2011;<br><br>2013	X	RCT	None	Brazil	University laboratory	Supervised exercise training	12 weeks	Aerobic exercise	3x / week	Doctoral-level physiotherapist	Not specified
14-18	Dantas;<br><br>Dantas;<br><br>Gil;<br><br>Gil;<br><br>Murai	2018;<br><br>2020;<br><br>2021A;<br><br>2021B;<br><br>2019	Dantas – X<br><br>Dantas – X<br><br>Gil – NO<br><br>Murai – NO	RCT	None	Brazil	University laboratory; hospital	Supervised exercise training	26 weeks	Aerobic exercise, resistance training	3x / week	Doctoral-level graduate student in exercise physiology	Not specified
19	Da Silva	2015	X	Case report	None	Brazil	Clinic; laboratory	Supervised exercise training	26 weeks	Resistance training exercises, aerobic exercise, flexibility training	3x/ week	Physical education teacher with specialization in exercise physiology	Doctoral level
20	de Oliveira	2016	X	RCT	None	Brazil	University hospital	Supervised exercise training	30 days	Customized program of walking, breathing exercises, some resistance exercises (1kg), and stretching	2x/ week	Physiotherapist	Not specified
21-22	Egberts;<br><br>Egberts	2010;<br><br>2011	X	RCT	None	Australia	University hospital	Supervised exercise training	12 weeks	Not specified	Not specified	Certified Personal Trainer (CPT)	Not specified

23	González -Cutre	201 9	X	Quali tative	None	Spain	University laboratory	Motivational physical activity program	26 weeks	Endurance training, resistance training with machines, games, directed activities, dance, body expression, core-training	2x/ week (Months 1 & 2); 3/ week (Months 3 & 4); 4x/ week (Months 5 & 6)	Sports science professional instructors	Master 's degree
24	Hanvold	201 9	X	RCT	None	Norway	University hospital	Group-based lifestyle intervention with supervised exercise training	2 years	Measurement s of body weight, lecture on given topic, group work and/or assignment, 30-minute supervised physical activity	16 group meetings over 2 years	Clinical dietitians	PhD and master s' studen ts
25	Hassann ejad	201 7	X	RCT	None	Iran	University laboratory	Unsupervised exercise training	12 weeks	Walking, resistance training	3-5x/ week (Weeks 5-12)	No supervision	Sports medici ne special ist
26	Herrera	202 0		RCT	None	Chile	Public hospital	Supervised exercise training	16 weeks	Moderate-intensity continuous training (MICT), high-intensity interval training (HIIT)	2x/ week	Physical therapist	Not specifi ed
27	Herring	201 7	X	RCT	None	United Kingdo m	University hospital; at home	Supervised exercise training	12 weeks	Aerobic exercise, resistance training	3x/ week	Exercise and sports science professional / REPS Gym instructor/ exercise physiologist	PhD physic al activity , lifestyl e and bariatri c surger y; MSc

													clinical exercise science; REPS
28	Jassil	2015	X	Pilot study	None	United Kingdom	University hospital; at home	Supervised exercise training; lifestyle education; nutritional/behavioral intervention	8 weeks	Circuit training	Weekly; 5x/ week	Physiotherapist,<br><br>Exercise specialist	Not specified
29	Jiménez-Loaisa	2020	X	Quasi-experimental	None	Spain	University public fitness center	Motivational physical activity intervention (MPAI)	26 weeks	Aerobic exercise, resistance training with machines, body expression, dance, directed activities, swimming, core training	2x/ week (Months 1 & 2); 3x/ week (Months 3 & 4); 4x/ week (Months 5 & 6)	Exercise and sport science professionals	Degree in sports science; master's degree in physical activity and health
30-31	Marc-Hernández ;<br><br>Marc-Hernández	2019;<br><br>2020	2019 – X<br><br>2020 – NO	Prospective intervention; RCT	EFIBAR RCT	Spain	University laboratory; university hospital	Supervised exercise training	12 weeks; 20 weeks	Endurance training, resistance training, HIIT training, flexibility training	3x/ week; 2x/ week (Weeks 1-4); 3x/ week (Weeks 5-8); 4x/ week (Weeks 9-16)	Supervised by undergraduate students in exercise science	PhD in exercise science created the program; not certified by major credentialing bodies
32	Marchesi	2015	X	Prospective interv	None	Italy	University hospital	Supervised and unsupervised exercise training	43 weeks	Indoor or outdoor road running	3x/ week	Sports physician (MD), CPT	Not specified

				ention									
33-34	Marcon; Marcon	2011; 2017	X	Case series; RCT	PESO III	Brazil	University; hospital	Supervised and unsupervised exercise training	26 weeks; 16 weeks	Aerobic exercise, walking, stretching	1x/ week; 2x/ week	PhD researcher/ physical education teacher	Masters/PhD level
35	Morana	2018	X	Pilot study	None	France	Physical therapy clinic	Supervised exercise training; functional rehabilitation	10 weeks	Endurance training, rowing, core exercises, proprioception work, coordination	2x/ week	Physiotherapist	Not specified
36-40	Mundbjerg; Mundbjerg; Stolberg; Stolberg; Stolberg	2018; 2018; 2018; 2018; 2018	X	RCT	None	Denmark	Public fitness center	Supervised exercise training	26 weeks	Endurance training, resistance training	2x/ week	Physiotherapist	Not specified
41	Muschitz	2015	X	RCT	BABS	Austria	University hospital	Unsupervised exercise training with diary monitoring	2 years	Nordic walking, strength perseverance, equipment training	3x/ week (Nordic walking), 2x/ week (strength perseverance, equipment training)	Physical medicine expert	Not specified
42	Ng	2015	X	Prospective intervention	LIFE	Singapore	Hospital	Semi-supervised exercise training	12 weeks	Not specified	Monthly	PhD physiotherapist/exercise physiologist, masters-level physiotherapist/exercise	All with bachelors degree (or higher) in physiotherap

												physiologist, therapy assistants	y. 3 with Masters. 1 with PhD.
43	Onofre	2017	X	Pilot study	None	Brazil	University laboratory	Supervised exercise training	12 weeks	Aerobic exercise, resistance training, stretching	3x/ week	Physiotherapist	Trained in cardiac and metabolic rehabilitation
43	Picó-Sirvent	2019	X	Pilot study	None	Spain	University laboratory	Supervised exercise training	26 weeks	Aerobic exercise, resistance training, HIIT training	2x/ week (Month 1), 3x/ week (Month 2), 4x/ week (Months 3-6)	Supervised undergraduate students in exercise science	Not specified
44	Proulx	2018	X	Prospective intervention	None	Canada	University fitness center	Supervised exercise training	12 weeks	Aerobic exercise, resistance training	3x/ week	Certified clinical exercise specialist	Not specified
46	Ricci	2020	X	RCT	None	Brazil	University laboratory	Supervised exercise training	6 weeks	Whole-body electromyostimulation, dynamic exercise	5x/ week	Physiotherapist	Not specified
47	Rojhani-Shirazi	2016	X	RCT	None	Iran	University laboratory	Supervised exercise training	4 weeks	Balance training	4x/ week	Physical therapist	Master's degree
48	Rothwell	2015	X	Retrospective review	BandFit	Australia	Private hospital, at home	Supervised and unsupervised exercise training	1 year; 3 years	"BandFit", a personal trainer-led education and participant exercise program	Variable (0, 1, or more than 1 sessions)	CPT	Not specified

49	Stegen	2011	X	Pilot study	None	Belgium	University hospital	Supervised exercise training	12 weeks	Endurance training, resistance training	3x/ week	Movement and rehabilitation expert	Master's degree
50	Soriano-Maldonado	2020	X	RCT	EMOVAR	Spain	University fitness center	Supervised exercise training	16 weeks	Aerobic exercise, resistance training	3x/ week	CPT	Grade in physical activity and sports sciences, and either a master's degree in personal training or >2 years' experience training obese people
51-52	Villa-González; Artero	2019; 2021	VG – X<br>Artero – NO	RCT	EFIBAR	Spain	University fitness center	Supervised exercise training	16 weeks	Aerobic exercise, resistance training	3x/ week	CPT	Bachelor's degree
53	Wiklund	2015	X	RCT	None	Sweden	University hospital	Supervised exercise training	1 week	Walking with step counter	Daily	Nurse,<br>Physiotherapist	Not specified

* Various trials/studies resulted in multiple publications, which were combined.

REFERENCES

Studies found initially (1-71) and added later (72-83). See results section of manuscript for more details.

1. Zagarins SE, Allen NA, Skinner SS, Kemper AJ, Welch G. Improved exercise behaviors associated with a comprehensive structured exercise program following bariatric surgery. Bariatric Nursing and Surgical Patient Care. 2011;6(2):85-90. doi: 10.1089/bar.2011.9971
2. Woodlief TL, Carnero EA, Standley RA, Distefano G, Anthony SJ, Dubis GS, et al. Dose response of exercise training following roux-en-Y gastric bypass surgery: A randomized trial. Obesity (Silver Spring). 2015;23(12):2454-61. doi: 10.1002/oby.21332
3. Wiklund M, Sundqvist E, Olsén MF. Physical Activity in the Immediate Postoperative Phase in Patients Undergoing Roux-en-Y Gastric Bypass-a Randomized Controlled Trial. Obesity Surgery. 2015;25(12):2245-50. doi: 10.1007/s11695-015-1690-y
4. Villa-González E, Barranco-Ruiz Y, Rodríguez-Pérez MA, Carretero-Ruiz A, García-Martínez JM, Hernández-Martínez A, et al. Supervised exercise following bariatric surgery in morbid obese adults: CERT-based exercise study protocol of the EFIBAR randomised controlled trial. BMC Surgery. 2019;19(1):127. doi: 10.1186/s12893-019-0566-9
5. Tardif I, Auclair A, Piché M-E, Biertho L, Marceau S, Hould F-S, et al. Impact of a 12-Week Randomized Exercise Training Program on Lipid Profile in Severely Obese Patients Following Bariatric Surgery. Obesity Surgery. 2020;30(8):3030-6. doi: 10.1007/s11695-020-04647-5
6. Stolberg CR, Mundbjerg LH, Funch-Jensen P, Gram B, Bladbjerg E-M, Juhl CB. Effects of gastric bypass surgery followed by supervised physical training on inflammation and endothelial function: A randomized controlled trial. Atherosclerosis. 2018;273:37-44. doi: 10.1016/j.atherosclerosis.2018.04.002
7. Stolberg CR, Mundbjerg LH, Funch-Jensen P, Gram B, Bladbjerg E-M, Juhl CB. Reply to: "Replicability of exercise programs following bariatric surgery". Atherosclerosis. 2018;278:332-3. doi: 10.1016/j.atherosclerosis.2018.09.028
8. Stolberg CR, Mundbjerg LH, Bladbjerg E-M, Funch-Jensen P, Gram B, Juhl CB. Physical training following gastric bypass: effects on physical activity and quality of life-a randomized controlled trial. Quality of Life Research. 2018;27(12):3113-22. doi: 10.1007/s11136-018-1938-9
9. Stegen S, Derave W, Calders P, Laethem CV, Pattyn P. Physical fitness in morbidly obese patients: effect of gastric bypass surgery and exercise training. Obesity Surgery. 2011;21(1):61-70. doi: 10.1007/s11695-009-0045-y
10. Soriano-Maldonado A, Martínez-Forte S, Ferrer-Márquez M, Martínez-Rosales E, Hernández-Martínez A, Carretero-Ruiz A, et al. Physical Exercise following bariatric surgery in women with Morbid obesity. Medicine (Baltimore). 2020;99(12). doi: 10.1097/MD.0000000000019427
11. Silva TLTBd, Fontes MT, Mariano RD, Mota MM. Effects of the program of supervised exercise on body composition after bariatric surgery: case report. Revista Brasileira de Obesidade, Nutrição e Emagrecimento. 2015;9(52). doi:
12. Shah M, Snell PG, Rao S, Adams-Huet B, Quittner C, Livingston EH, et al. High-volume exercise program in obese bariatric surgery patients: a randomized, controlled trial. Obesity (Silver Spring). 2011;19(9):1826-34. doi: 10.1038/oby.2011.172
13. Rothwell L, Lilian Kow JT. Effect of a post-operative structured exercise programme on short-term weight loss after obesity surgery using adjustable gastric bands. Obesity Surgery. 2015;25(1):126-8. doi: 10.1007/s11695-014-1323-x
14. Rojhani-Shirazi Z, Mansoriyan SA, Hosseini SV. The effect of balance training on clinical balance performance in obese patients aged 20–50 years old undergoing sleeve gastrectomy. European Surgery. 2016;48:105-9. doi: 10.1007/s10353-015-0379-8
15. Ricci PA, Thommazo-Luporini LD, Jürgensen SP, André LD, Haddad GF, Arena R, et al. Effects of Whole-Body Electromyostimulation Associated with Dynamic Exercise on Functional Capacity and Heart Rate Variability After Bariatric Surgery: a Randomized, Double-Blind, and Sham-Controlled Trial. Obesity Surgery. 2020;30(10):3862-71. doi: 10.1007/s11695-020-04724-9
16. Proulx É, Auclair A, Piché M-E, Harvey J, Pettigrew M, Biertho L, et al. Safety of Blood Glucose Response Following Exercise Training After Bariatric Surgery. Obesity Surgery. 2018;28(12):3976-83. doi: 10.1007/s11695-018-3449-8
17. Powell SM, Fasczewski KS, Gill DL, Davis PG. Go with the FLOW: Implementation of a psychological skills intervention in an exercise program for post-bariatric surgery patients. Journal of Health Psychology. 2020;25(13-14):2260-71. doi: 10.1177/1359105318793182

18. Powell SM, Fasczewski KS, Gill DL, Davis PG. Go with the FLOW: Implementation of a psychological skills intervention in an exercise program for post-bariatric surgery patients. Journal of Health Psychology. 2018:1-12. doi: 10.1177/1359105318793182
19. Picó-Sirvent I, Aracil-Marco A, Pastor D, Moya-Ramón M. Effects of a Combined High-Intensity Interval Training and Resistance Training Program in Patients Awaiting Bariatric Surgery: A Pilot Study. Sports (Basel). 2019;7(3):72. doi: 10.3390/sports7030072
20. Parikh M, Dasari M, McMacken M, Ren C, Fielding G, Ogedegbe G. Does a preoperative medically supervised weight loss program iimprove bariatric surgery outcomes? A pilot randomized study. Surgical Endoscopy. 2012;26:853-61. doi: 10.1007/s00464-011-1966-9
21. Onofre T, Carlos R, Oliver N, Felismino A, Fialho D, Corte R, et al. Effects of a Physical Activity Program on Cardiorespiratory Fitness and Pulmonary Function in Obese Women after Bariatric Surgery: a Pilot Study. Obesity Surgery. 2017;27(8):2026-33. doi: 10.1007/s11695-017-2584-y
22. Oliveira JJJd, Freitas ACTd, Almeida AAd. Postoperative effect of physical therapy related to functioncal capacity and respiratory muscle strength in patients submitted to bariatric surgery. Arquivox Brasileios de Cirurgia Digestiva. 2016;28:43-7. doi: 10.1590/0102-6720201600S10012
23. Ng DP, McClements KE, Yeo A, Chen X, Png EK, Ng C. Efficacy of supervised exercise training in patients post bariatric surgery. Physiotherapy. 2015;101:E1083-E4. doi: https://doi.org/10.1016/j.physio.2015.03.1973
24. Muschitz C, Kocijan R, Haschka J, Zendeli A, Pirker T, Geiger C, et al. The Impact of Vitamin D, Calcium, Protein Supplementation, and Physical Exercise on Bone Metabolism After Bariatric Surgery: The BABS Study Journal of Bone and Mineral Research. 2016;31(3):672-82. doi: 10.1002/jbmr.2707
25. Mundbjerg LH, Stolberg CR, Cecere S, Bladbjerg E-M, Funch-Jensen P, Gram B, et al. Supervised Physical Training Improves Weight Loss After Roux-en-Y Gastric Bypass Surgery: A Randomized Controlled Trial. Obesity (Silver Spring). 2018;26(5):828-37. doi: 10.1002/oby.22143
26. Mundbjerg LH, Stolberg CR, Bladbjerg E-M, Funch-Jensen P, Juhl CB, Gram B. Effects of 6 months supervised physical training on muscle strength and aerobic capacity in patients undergoing Roux-en-Y gastric bypass surgery: a randomized controlled trial. Clinical Obesity. 2018;8(4):227-35. doi: 10.1111/cob.12256
27. Morana C, Collignon M, Nocca D. Effectiveness of a Functional Rehabilitation Program After Bariatric Surgery: a Pilot Study. Obesity Surgery. 2018;28(8):2321-6. doi: 10.1007/s11695-018-3154-7
28. Marcon ER, Gus I, Neumann CR. Impact of a minimum program of supervised exercises in the cardiometabolic risk in patients with morbid obesity. Arquivox Brasileios de Endocrinologia e Metabologia. 2011;55(5):331-8. doi: 10.1590/s0004-27302011000500006
29. Marcon ER, Baglioni S, Bittencourt L, Lopes CLN, Neumann CR, Trindade MRM. What Is the Best Treatment before Bariatric Surgery? Exercise, Exercise and Group Therapy, or Conventional Waiting: a Randomized Controlled Trial. Obesity Surgery. 2017;27:763-73. doi: 10.1007/s11695-016-2365-z
30. Marchesi F, Sario GD, Reggiani V, Tartamella F, Giammaresi A, Cecchini S, et al. Road Running After Gastric Bypass for Morbid Obesity: Rationale and Results of a New Protocol. Obesity Surgery. 2015;25(7):1162-70. doi: 10.1007/s11695-014-1517-2
31. Marc-Hernández A, Ruiz-Tovar J, Aracil A, Guillén S, Moya-Ramón M. Impact of Exercise on Body Composition and Cardiometabolic Risk Factors in Patients Awaiting Bariatric Surgery. Obesity Surgery. 2019;29(12):3891-900. doi: 10.1007/s11695-019-04088-9
32. Lopez YON, Coen PM, GoodpasteR BH, Seyhan AA. Gastric bypass surgery with exercise alters plasma microRNAs that predict improvements in cardiometabolic risk. International Journal of Obesity. 2017;41(7):1121-30. doi: 10.1038/ijo.2017.84
33. Klasnja P, Rosenberg DE, Zhou J, Anau J, Gupta A, Arterburn DE. A quality-improvement optimization pilot of BariFit, a mobile health intervention to promote physical activity after bariatric surgery. Translational Behavioral Medicine. 2020:1-10. doi: 10.1093/tbm/ibaa040
34. Kerrigan DJ, Loduca-Duhaime A, Carlin AM, Szymanski W, Genaw J. A trans-disciplinary approach to weight loss inthe super super morbidly obese. Journal of Clinical Exercise Physiology. 2012;1(1):30-4. doi:
35. Kelley W. Medically integrated exercise: Florida hospital celebration health bariatric program. ACSM's Health & Fitness Journal. 2006;10(6):28-30. doi:
36. Kalarchian MA, Marcus MD, Courcoulas AP, Cheng Y, Levine MD. Preoperative lifestyle intervention in bariatric surgery: initial results from a randomized, controlled trial. Obesity (Silver Spring). 2013;21(2):254-60. doi: 10.1002/oby.20069

37. Jiménez-Loaisa A, González-Cutre D, Beltrán-Carrillo VJ, Alcaraz-Ibáñez M. Changes in Bariatric Patients' Physical Activity Levels and Health-Related Quality of Life Following a Postoperative Motivational Physical Activity Intervention. Obesity Surgery. 2020;30(6):2302-12. doi: 10.1007/s11695-020-04489-1.
38. Jassil FC, Manning S, Lewis N, Steinmo S, Kingett H, Lough F, et al. Feasibility and Impact of a Combined Supervised Exercise and Nutritional-Behavioral Intervention following Bariatric Surgery: A Pilot Study. Journal of Obesity. 2015;2015:12. doi: https://doi.org/10.1155/2015/693829
39. Huck CJ. Effects of supervised resistance training on fitness and functional strength in patients succeeding bariatric surgery. Journal of Strength and Conditioning Research. 2015;29(3):589-95. doi: 10.1519/JSC.0000000000000667
40. Hickey MS, Gavigan KE, McCammon MR, Tyndhall GL, Pories WJ, Israel RG, et al. Effects of 7 days of exercise training on insulin action in morbidly obese men. Clinical Exercise Physiology. 1999;1(1):24-8. doi:
41. Herring L, Stevinson C, Carter P, Biddle S, Bowrey D, Sutton C, et al. The effects of supervised exercise training 12-24 months after bariatric surgery on physical function and body composition: a randomised controlled trial. International Journal of Obesity. 2017;41:909-16. doi:
42. Hassannejad A, Khalaj A, Mansournia MA, Tabesh MR, Alizadeh Z. The Effect of Aerobic or Aerobic-Strength Exercise on Body Composition and Functional Capacity in Patients with BMI ≥35 after Bariatric Surgery: a Randomized Control Trial. Obesity Surgery. 2017;27(11):2792-801. doi: 10.1007/s11695-017-2717-3
43. Hanvold SE, Vinknes KJ, Løken EB, Hjartåker A, Klungsøyr O, Birkeland E, et al. Does Lifestyle Intervention After Gastric Bypass Surgery Prevent Weight Regain? A Randomized Clinical Trial. Obesity Surgery. 2019;29(11). doi: 10.1007/s11695-019-04109-7
44. Hamdy O, Ashrafzadeh S, Mottalib A. Weight Management in Patients with Type 2 Diabetes: a Multidisciplinary Real-world Approach. Current Diabetes Reports. 2018;18(9). doi: 10.1007/s11892-018-1030-4
45. Halperin F, Ding S-A, Simonson DC, Panosian J, Goebel-Fabbri A, Wewalka M, et al. Roux-en-Y gastric bypass surgery or lifestyle with intensive medical management in patients with type 2 diabetes: feasibility and 1-year results of a randomized clinical trial. JAMA Surgery. 2014;149(7):716-26. doi: 10.1001/jamasurg.2014.514
46. Gonalez-Cutre D, Jimenez-Loaisa A, Romero-Elias M, Beltran-Carrillo VJ. Exploring bariatric patients' need for novelty in a motivational physical activity program: a qualitative study. European Journal of Human Movement. 2019;43:1-12. doi:
47. Gill DL, Fasczewski KS, Reifsteck EJ, Rothberger SM, Davis PG. Evaluation of an exercise programme for post-bariatric surgery patients: views of participants. Obesity Science & Practice. 2018;4(3):259-67. doi: 10.1002/osp4.165
48. Funderburk JA, Callis S. Aquatic intervention effect on the quality of life prior to obesity surgery: a pilot study. Annual in Therapeutic Recreation; 2010.
49. Egberts KJ, Brown W, O'Brien PE. Optimising lifestyle factors to achieve weight loss in surgical patients. Obesity Research & Clinical Practice. 2010;4:S13. doi: https://doi.org/10.1016/j.orcp.2010.09.026
50. Egberts K, Brown W, O'Brien P. Optimising lifestyle factors to achieve weight loss in surgical patients. Surgery for Obesity and Related Diseases. 2011;7:368. doi:
51. Dantas WS, Roschel H, Murai IH, Gil S, Davuluri G, Axelrod CL, et al. Exercise-Induced Increases in Insulin Sensitivity After Bariatric Surgery Are Mediated By Muscle Extracellular Matrix Remodeling. Diabetes. 2020;69(8):1675-91. doi: 10.2337/db19-1180
52. Dantas WS, Gil S, Murai IH, Costa-Hong V, Peçanha T, Merege-Filho CAA, et al. Reversal of Improved Endothelial Function After Bariatric Surgery Is Mitigated by Exercise Training. Journal of the American College of Cardiology. 2018;72(18):2278-9. doi: 10.1016/j.jacc.2018.07.094
53. Daniels P, Burns RD, Brusseau TA, Hall MS, Davidson L, Adams TD, et al. Effect of a randomised 12-week resistance training programme on muscular strength, cross-sectional area and muscle quality in women having undergone Roux-en-Y gastric bypass. Journal of Sports Sciences. 2018;36(5):529-35. doi: 10.1080/02640414.2017.1322217
54. Creel DB, Schuh LM, Reed CA, Gomez AR, Hurst LA, Stote J, et al. A randomized trial comparing two interventions to increase physical activity among patients undergoing bariatric surgery. Obesity (Silver Spring). 2016;24(8):1660-8. doi: 0.1002/oby.21548
55. Coen PM, Tanner CJ, Helblin NL, Dubis GS, Hames KC, Xie H, et al. Clinical trial demonstrates exercise following bariatric surgery improves insulin sensitivity. Journal of Clinical Investigation. 2015;125(1):248-57. doi: 10.1172/JCI78016

56. Coen PM, Menshikova EV, Distefano G, Zheng D, Tanner CJ, Standley RA, et al. Exercise and Weight Loss Improve Muscle Mitochondrial Respiration, Lipid Partitioning, and Insulin Sensitivity After Gastric Bypass Surgery. Diabetes. 2015;64(11):3737-50. doi: 10.2337/db15-0809
57. Castello-Simões V, Simões RP, Beltrame T, Bassi D, Catai AM, Arena R, et al. Effects of aerobic exercise training on variability and heart rate kinetic during submaximal exercise after gastric bypass surgery – a randomized controlled trial. Disability and Rehabilitation. 2013;35(4):334-42. doi: 10.3109/09638288.2012.694575
58. Castello V, Simões RP, Bassi D, Catai AM, Arena R, Borghi-Silva A. Impact of aerobic exercise training on heart rate variability and functional capacity in obese women after gastric bypass surgery. Obesity Surgery. 2011;21(11):1739-49. doi: 10.1007/s11695-010-0319-4
59. Carnero EA, Dubis GS, Hames KC, Jakicic JM, Houmard JA, Coen PM, et al. Randomized trial reveals that physical activity and energy expenditure are associated with weight and body composition after RYGB. Obesity (Silver Spring). 2017;25(7):1206-16. doi: 10.1002/oby.21864
60. Campanha-Versiani L, Pereira DAG, Ribeiro-Samora GA, Ramos AV, Diniz MFHdS, Marco LAD, et al. The Effect of a Muscle Weight-Bearing and Aerobic Exercise Program on the Body Composition, Muscular Strength, Biochemical Markers, and Bone Mass of Obese Patients Who Have Undergone Gastric Bypass Surgery. Obesity Surgery. 2017;27(8):2129-37. doi: 10.1007/s11695-017-2618-5
61. Brown WA, Burton PR, Shaw K, Smith B, Maffescioni S, Comitti B, et al. A Pre-Hospital Patient Education Program Improves Outcomes of Bariatric Surgery. Obesity Surgery. 2016;26(9):2074-81. doi: 10.1007/s11695-016-2075-6
62. Brandenburg D, Kotlowski R. Practice makes perfect? Patient response to a prebariatric surgery behavior modification program. Obesity Surgery. 2005;15(1):125-32. doi: 10.1381/0960892052993594
63. Bond DS, Vithiananthan S, Thomas JG, Trautvetter J, Unick JL, Jakicic JM, et al. Bari-Active: a randomized controlled trial of a preoperative intervention to increase physical activity in bariatric surgery patients. Surgery for Obesity and Related Diseases. 2015;11(1):169-77. doi: 10.1016/j.soard.2014.07.010
64. Bond DS, Thomas JG, King WC, Vithiananthan S, Trautvetter J, Unick JL, et al. Exercise improves quality of life in bariatric surgery candidates: results from the Bari-Active trial. Obesity Journal. 2015;23(3):536-42. doi: 10.1002/oby.20988
65. Bond D, Thomas J, Vithiananthan S, Unick J, Webster J, Roye G, et al. Intervention-related increases in preoperative physical activity are maintained 6-months after Bariatric surgery: results from the bari-active trial. International Journal of Obesity. 2017;41:467-70. doi: 10.1038/ijo.2016.237
66. Berggren JR, Boyle KE, Chapman WH, Houmard JA. Skeletal muscle lipid oxidation and obesity: influence of weight loss and exercise. Am J Physiol Endocrinol Metab. 2008(294):E726-E32. doi: 10.1152/ajpendo.00354.2007
67. Baillot A, Vallée C-A, Mampuya WM, Dionne IJ, Comeau E, Méziat-Burdin A, et al. Effects of a Pre-surgery Supervised Exercise Training 1 Year After Bariatric Surgery: a Randomized Controlled Study. Obesity Surgery. 2018;28(4):955-62. doi: 10.1007/s11695-017-2943-8
68. Baillot A, Mampuya WM, Dionne sJ, Comeau E, Méziat-Burdin A, Langlois M-F. Impacts of Supervised Exercise Training in Addition to Interdisciplinary Lifestyle Management in Subjects Awaiting Bariatric Surgery: a Randomized Controlled Study. Obesity Surgery. 2016;26(11):2602-10. doi: 10.1007/s11695-016-2153-9
69. Baillot A, Mampuya WM, Comeau E, Méziat-Burdin A, Langlois MF. Feasibility and impacts of supervised exercise training in subjects with obesity awaiting bariatric surgery: a pilot study. Obesity Surgery. 2013;23(7):882-91. doi: 10.1007/s11695-013-0875-5
70. Baillot A, Boissy P, Tousignant M, Langlois M-F. Feasibility and effect of in-home physical exercise training delivered via telehealth before bariatric surgery. Journal of Telemedicine and Telecare. 2016;23(5):529-35. doi: 10.1177/1357633X16653511
71. Audrey Auclair, Harvey J, Leclerc J, Pich M-E, Kim O'Connor M, Nadreau É, et al. Determinants of Cardiorespiratory Fitness After Bariatric Surgery: Insights From a Randomised Controlled Trial of a Supervised Training Program. Canadian Journal of Cardiology. 2020;37(2):251-9. doi: 10.1016/j.cjca.2020.03.032
72. Coleman KJ, Caparosa SL, Nichols JF, Fujioka K, Koebnick C, McCloskey KN, et al. Understanding the Capacity for Exercise in Post-Bariatric Patients. Obesity Surgery. 2017;27(1):51-8. doi: 10.1007/s11695-016-2240-y
73. Gilbertson NM, Gaitán JM, Osinski V, Rexrode EA, Garmey JC, Mehaffey JH, et al. Pre-operative aerobic exercise on metabolic health and surgical outcomes in patients receiving bariatric surgery: A pilot trial. PLoS One. 2020;15(10). doi: 10.1371/journal.pone.0239130

74. Panosian J, Ding S-A, Wewalka M, Simonson DC, Goebel-Fabbri A, Foster K, et al. Physical activity in obese type 2 diabetes after gastric bypass or medical management. The American Journal of Medicine. 2017;130(1):83-92. doi: 10.1016/j.amjmed.2016.07.019
75. Lemieux-Simard C, Pettigrew M, Auclair A, Piche ME, Biertho L, Marceau S, et al. Impact of a 12-week supervised exercise program on weight loss trajectory, daily physical activity levels and sedentary behaviors after bariatric surgery. European Heart Journal. 2021;42. doi: 10.1093/eurheartj/ehab724.2605
76. Pettigrew M, Auclair A, Harvey J, Marceau S, Biertho L, Poirier P. Daily physical activity level after bariatric surgery and a 12-week supervised exercise program: Are they active enough? Canadian Journal of Cardiology. 2019;35(10). doi:
77. Brun J-F, Lasteyrie V, Hammoudi L, Nocca D, Ghanassia E, Noirez P, et al. Exercise targeted at the level of maximal lipid oxidation (LIPOXmax) improves weight loss, decreases orexigenic pulsions and increases satiety after sleeve gastrectomy. Global Journal of Obesity, Diabetes and Metabolic Syndrome. 2019;6(2):17-21. doi: 10.17352/2455-8583.000037
78. Gil S, Kirwan JP, Murai IH, Dantas WS, Merege-Filho CAA, Ghosh S, et al. A randomized clinical trial on the effects of exercise on muscle remodelling following bariatric surgery. Journal of Cachexia, Sarcopenia, and Muscle. 2021;12(6):1440-55. doi: 10.1002/jcsm.12815
79. Gil S, Peçanha T, Dantas WS, Murai IH, Merege-Filho CAA, Sá-Pinto ALd, et al. Exercise Enhances the Effect of Bariatric Surgery in Markers of Cardiac Autonomic Function. Obesity Surgery. 2021;31(3):1381-6. doi: 10.1007/s11695-020-05053-7
80. Murai IH, Roschel H, Dantas WS, Gil S, Merege-Filho C, Cleva Rd, et al. Exercise Mitigates Bone Loss in Women With Severe Obesity After Roux-en-Y Gastric Bypass: A Randomized Controlled Trial. Journal of Clinical Endocrinology and Metabolism. 2019;104(10):4639-50. doi: 10.1210/jc.2019-00074
81. Herrera A, Tabach A, Andaur K, Zamuner A. Effect of physical exercise in bariatric surgery patients: protocol of a randomized controlled clinical trial. Trials. 2020;22. doi: https://doi.org/10.1186/s13063-021-05056-4
82. Marc-Hernández A, Ruiz-Tovar J, Aracil A, Guillén S, Moya-Ramón M. Effects of a High-Intensity Exercise Program on Weight Regain and Cardio-metabolic Profile after 3 Years of Bariatric Surgery: A Randomized Trial. Scientific Reports. 2020;10(1):3123. doi: 10.1038/s41598-020-60044-z
83. Artero EG, Ferrez-Márquez M, Torrente-Sánchez MJ, Martínez-Rosales E, Carretero-Ruiz A, Hernández-Martínez A, et al. Supervised Exercise Immediately After Bariatric Surgery: the Study Protocol of the EFIBAR Randomized Controlled Trial. Obesity Surgery. 2021;31(10):4227-35. doi: 10.1007/s11695-021-05559-8
