# Supplementary material for: Role of the exercise professional in metabolic and bariatric surgery
Source: Surg Obes Relat Dis. Author manuscript; Available in PMC 2025 Jan 1. (PMC11311246; doi:10.1016/j.soard.2023.09.026)
Supplement: Supplement 7 [file NIHMS2008743-supplement-Supplement_7.pdf]

Supplement 7. Summary of bin sort per analyst.										
**Rater #**	**1**	**2**	**3**	**4**	**5**	**6**	**7**	**8**	**9**	**10**	**Average**
**Analyst initials**	**DB**	**CG**	**EB**	**BG**	**MSK**	**LR**	**JH**	**LH**	**RM**	**MD**	
**Bins completed (#)**	12	14	10	11	14	11	10	11	10	15	11.8
**Bins w/ 8 items**	0	1	1	0	0	0	1	0	0	0	0.3
**Bins w/ 7 items**	0	1	1	0	0	0	1	0	0	0	0.3
**Bins w/ 6 items**	0	0	0	1	0	3	1	3	2	1	1.1
**Bins w/ 5 items**	5	0	1	2	1	0	1	2	3	1	1.6
**Bins w/ 4 items**	1	1	2	2	2	2	1	0	2	4	1.7
**Bins w/ 3 items**	3	5	3	1	5	4	2	2	2	1	2.8
**Bins w/ 2 items**	1	3	2	2	4	0	1	3	1	5	2.2
**Bins w/ 1 items**	2	3	0	3	1	2	2	1	0	3	1.7
**Items to discard**	0	0	2	7	3	2	2	2	0	0	1.8
**Ave items/bin***	3.5	3.1	4.1	3.3	2.9	3.7	4.1	3.7	4.3	2.9	3.6
**Items missing to add**	0	14	0	7	0	5	6	0	2	0	3.4
* ([# items – discarded items]/bins completed)											
