# Supplementary material for: Role of the exercise professional in metabolic and bariatric surgery
Source: Surg Obes Relat Dis. Author manuscript; Available in PMC 2025 Jan 1. (PMC11311246; doi:10.1016/j.soard.2023.09.026)
Supplement: Supplement 9 [file NIHMS2008743-supplement-Supplement_9.pdf]

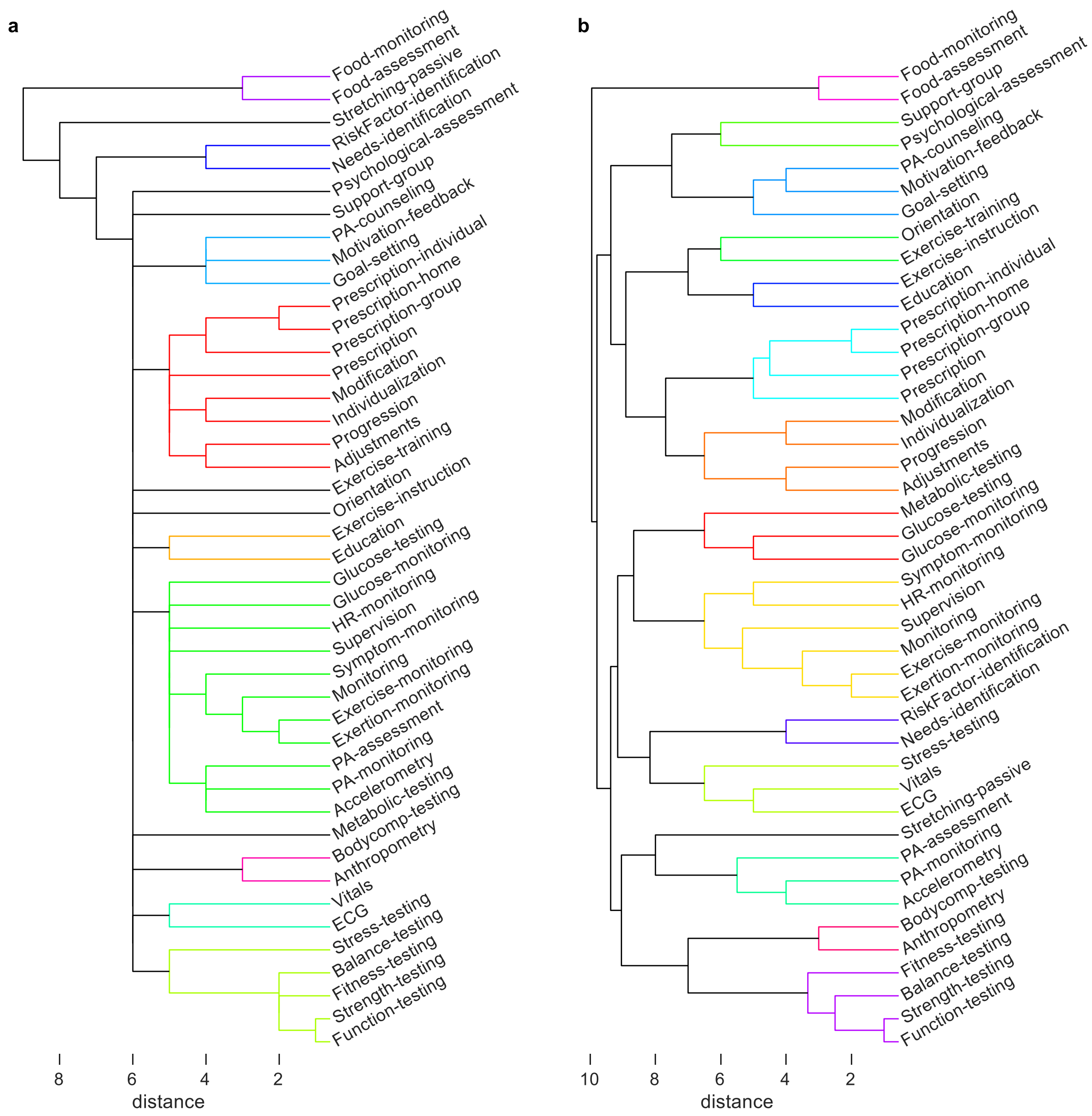

Supplement 9. Alternative models (A and B) generated by the hierarchical cluster analysis and deliberated in the Delphi process.
