# Supplementary material for: Role of the exercise professional in metabolic and bariatric surgery
Source: Surg Obes Relat Dis. Author manuscript; Available in PMC 2025 Jan 1. (PMC11311246; doi:10.1016/j.soard.2023.09.026)
Supplement: Supplement 12 [file NIHMS2008743-supplement-Supplement_12.pdf]

Supplement 12a. Patient-care-related tasks completed by the exercise professional for each study: Recode and re-review for 14 higher-order themes (HOTs) within 7 super higher-order themes (SHOTs) [1, 2, 3]

	EXERCISE-RELATED HEALTH ASSESSMENT		BODY COMPOSITION AND PHYSICAL FITNESS ASSESSMENT		LIFESTYLE PHYSICAL AND SEDENTARY BEHAVIOR ASSESSMENT	EDUCATION, INSTRUCTION, AND PRESCRIPTION				EXERCISE MONITORING		BEHAVIORAL COUNSELING AND PSYCHOSOCIAL SUPPORT		DIETARY SUPPORT
**Citation; (+) primary citation for study series**	**1- Risk factor and needs**	**2- Exercise-Related Health Assessment**	**3. Body Composition Assessment**	**4. Fitness Testing**	**5. Lifestyle physical activity and sedentary behavior assessment**	**6. Exercise and Fitness Education**	**7. Physical Activity instruction**	**8. Exercise Prescription**	**9. Personalization**	**10. Metabolic Assessment**	**11. Monitoring**	**12. Counseling Techniques**	**13. Psychosocial Support**	**14. Dietary Support**
Auclair, 2020<br>Lemiux, 2021<br>Simard, 2019<br>Pettigrew, 2020<br>Tardif, 2020	X	X	X	X			X	X		X	X			
Berggren, 2008	/	X	X	X			X-1:1	X-1:1		X	X			X
Bond, 2015a<br>Bond, 2015 b<br>Bond, 2017	X	X	X	X		X-1:1i	X:1-1i	X-H	X		X	X		
Carnero, 2017<br>Coen, 2015a<br>Coen, 2015b<br>Lopez, 2017<br>Woodlief, 2015	X	X	X	X	X			X	X	X	X			
Daniels, 2018	X	X	X	X		X		X:1		X	X	X	X	
Dantas, 2020<br>Dantas, 2018<br>Gil, 2021a<br>Gil, 2021b<br>Murai, 2019	X	X	X	X	X		X	X		X	X			

Gill, 2018<br>Powell, 2018<br>Powell, 2020	**X**	**X**	**X**	**X**	**X**	**X**	**X**	**X-G**			**X**	**X**		**X**
Halparin, 2014a<br>Halparin, 2015b<br>Panosian, 2017<br>Hamdy, 2018	**X**	**X**	**X**	**/**	**X**	**X**	**/**	**X-G / X-1:1i**	**X**	**X**	**X**	**X**	**X**	**X**
Hickey, 1999	**X**	**X**	**X**	**X**	**/**	**X**	**/**	**X-1:1i**		**X**	**X**			
Huck, 2015	**X**	**X**	**X**	**X**	**X**		**X**	**X**		**X**	**X**	**X**		
Kelley, 2006	**X**	**X**	**X**	**X**	**X**	**X**	**/**	**X**	**X**	**X**	**X**	**/**		**X**
Kerrigan, 2012	**X**	**X**	**X**	**X**	**X**	**X**	**/**	**X-1:1i**	**X**		**X**	**X**		**X**
Morana, 2018	**X**	**X**	**X**	**X**	**X**			**X:1-1i**	**X**	**X**	**X**			
Shah, 2011	**X**	**X**	**X**	**X**	**X**	**X**		**X / X-1:1i**		**X**	**X**	**X**		**X**
Zagarins, 2011	**X**	**X**	**/**	**X**	**X**	**X**	**X**	**X-1:1**	**X**		**X**	**X**		**X**
	**15/15; 100%**	**15/15; 100%**	**15/15; 100%**	**15/15; 100%**	**11/15; 73%**	**9/15; 60%**	**11/15; 73%**	**15/15; 100%**	**7/15; 47%**	**11/15; 73%**	**15/15; 100%**	**9/15; 60%**	**2/15; 13%**	**7/15; 47%**
Castello, 2011<br>Castello-Simoes, 2013	**X**	**X**	**X**	**X**	**/**		**X**	**X: 1-1i**	**X**	**X**	**X**			
de Oliveira, 2016	**X**	**X**	**X**	**X**			**X**	**X:1-1I / X:Hi**			**X**	**X**		
Herrera, 2020	**X**	**X**	**X**	**X**	**X**	**X**	**X**	**X-G**		**X**	**X**			
Jassil, 2015	**X**	**/**	**X**	**/**	**X**	**X**		**X**	**X**	**X**	**X**	**X**		**X**
Morana, 2018	**X**	**X**	**X**	**X**	**X**			**X:1-1i**	**X**	**X**	**X**			
Mundbjerg, 2018<br>Mundbjerg, 2018<br>Stolberg, 2018<br>Stolberg, 2018	**X**		**X**			**X**	**/**	**X**	**X**	**X**	**X**	**X**		**X**

Stolberg, 2018														
Onofre, 2017	**X**	**X**	**X**	**X**				**X**	**X**	**X**	**X**			
Rojhani-Shirazi, 2016	X	/	X	X	X			/			X			
Ricci, 2020	**X**	**X**	**X**	**X**			**X**	**X**			**X**			
Wiklund, 2015	**X**	**/**	**X**		**X**	**X**		**/**	**X**		**X**			
	**10/10; 100%**	**9/10; 90%**	**10/10; 100%**	**8/10; 80%**	**6/10; 60%**	**4/10; 40%**	**5/10; 50%**	**10/10; 100%**	**6/10; 60%**	**6/10; 60%**	**10/10; 10%**	**3/10; 30%**	**0/10; 0%**	**2/10; 20%**
Baillot, 2013<br>Baillot, 2016<br>Baillot, 2016<br>Baillot, 2016	**X**	**X**	**X**	**X**	**X**			**X-G / X-1:1**	**X**	**X**	**X**	**X**		**X**
Brandonberg, 2005	**X**	**/**	**X**		**X**	**/**	**X**				**X**	**X**		**X**
Brown, 2016	**X**		**X**									**X**		**X**
Brun, 2019	**X**	**X**	**X**	**X**				**X**	**X**	**X**				
Campanha-Versiani 2017	**X**	**/**	**X**	**X**	**/**			**X**	**X**	**X**	**X**			**X**
Coleman, 2016	**X**	**X**	**X**	**X**			**X**	**X**			**X**			
Creel, 2016	**X**	**X**	**X**	**X**	**X**			**X**			**X**	**X**		**X**
DaSilva, 2015								**X**						
Egberts, 2010<br>Egberts, 2011	**X**		**/**							**X**	**/**			
Funderburk, 2010	**X**	**X**		**X**	**X**			**X-G**		**X**		**X**		
Gilbertson, 2020	**X**	**X**	**X**	**X**				**X-H**		**X**	**X**	**X**		**X**

Gonzalez-Cutre, 2020	**X**		**X**		**X**		**X**	**X**			**X**	**X**		
Hanvold, 2019	**X**	**X**	**X**		**X**			**X**		**X**	**X**	**X**	**X**	**X**
Hassannejad, 2017	**X**	**X**	**X**	**X**	**X**		**X**	**X**			**X**	**X**		**X**
Herring, 2017	**X**	**X**	**X**	**X**	**X**		**X**	**X-G**	**X**	**X**	**X**	**X**		**X**
Huck, 2015	**X**	**X**	**X**	**X**	**X**		**X**	**X-1:1i**	**X**	**X**	**X**	**X**		**X**
Jimenez-Loasia, 2020	**X**	**X**	**X**	**X**	**X**	**X**	**X**				**X**			
Kalarchian, 2013	**X**	**X**	**X**		**X**			**X**			**X**	**X**		**X**
Klasnja, 2020	**X**	***/***	**X**		***/***						**X**	**X**		**X**
Marc-Hernandez, 2019<br>Marc-Hernandez 2020	**X**	**X**	**X**	**X**	**X**	**X**	**X**	**X**		**X**	**X**			
Marchesi, 2015	**X**	**X**	**X**	**X**			**X**	**X**	**X**	**X**	**X**	**X**	**X**	**X**
Muschitz, 2015	**X**	**X**	**X**			**X**	**X**	**X**	**X**	**X**	**X**	**X**		**X**
Parikh, 2012	**X**	**X**	**X**		**X**	**X**		**X**		**X**	**X**	**X**	**X**	**X**
Pico-Sirvent, 2019	**X**	**X**	**X**	**X**			**X**	**X**	**X**	**X**	**X**			**X**
Proulx, 2018	**X**		**X**		**X**			**X**	**X**	**X**	**X**	**X**		**X**
Rothwell, 2015	**X**		**X**			**X**	**X**	**X-H**			**X**			
Soriano-Maldonado, 2020	**X**	**X**	**X**	**X**	**X**			**X**		**X**	**X**			**X**
Stegen, 2011	**X**	**X**	**X**	**X**			**X**	**X**	**X**	**X**	**X**			
Villa-González, 2019<br><br>Artero, 2021	**X**		**X**				**X**	**X**	**X**	**X**	**X**	**X**		**X**

	28/29; 97%	22/29; 76%	27/29; 93%	16/29; 55%	17/29; 59%	6/29; 21%	14/29; 48%	24/29; 83%	11/29; 38%	18/29; 62%	25/29; 86%	18/29; 62%	3/29; 10%	19/29; 34%
GRAND TOTAL	53/54; 98%	46/54; 85%	52/54; 96%	39/54; 72%	34/54; 63%	19/54; 35%	30/54; 56%	49/54; 91%	24/54; 44%	35/54; 65%	50/54; 93%	30/54; 56%	5/54; 9%	28/54; 44%

[1]: **Hamdy (2018) was not included because it was a review paper.**

[2]: **See Table 2b for coding**

[3]: **GREEN = EX PHYSIOLOGISTS; PHYSIOTHERAPISTS IN PEACH;**

Supplement 12b - Next page

Supplement 12b: Coding for Table 2a

	X: Explicitly stated<br><br>/: Implied	Setting	Standardized vs Individualized	Example format	Note
X-G	Explicitly stated;	Group setting	Standardized, by definition!	classroom setting; clinic; hospital	
X-1:1	Explicitly stated;	1-on-1 training	Standardized	Exam room at clinic, hospital recovery	
X-1:1i	Explicitly stated;	1-on-1 training	Individualized	Exam room at clinic, hospital recovery	
X-O	Explicitly stated	Online (independent- without the exercise professional present)	Standardized	Video tutorials	
X-Oi	Explicitly stated	Online	Individualized	Interactive/automated digital systems	None of the studies may have this
X-H	Explicitly stated	Home (independent- without the exercise professional present)	Standardized	Ex Rx they are given to do at home, same for everyone (similar to InfoSheet, below)	
X-Hi	Explicitly stated	Home (independent- without the exercise professional present)	Individualized	Home Exercise Program (HEP) given at discharge, similar to what physical therapists give	
X-T	Explicitly stated	telephone or video-conferencing	Standardized	Zoom group	
X-Ti	Explicitly stated	telephone or video-conferencing	individualized	Zoom one-on-one	
X-IS	Explicitly stated	Info sheet	Standardized, by definition!	Just informational, not a program, per se, like above.	
