# Supplementary material for: Role of the exercise professional in metabolic and bariatric surgery
Source: Surg Obes Relat Dis. Author manuscript; Available in PMC 2025 Jan 1. (PMC11311246; doi:10.1016/j.soard.2023.09.026)
Supplement: Supplement 11 [file NIHMS2008743-supplement-Supplement_11.pdf]

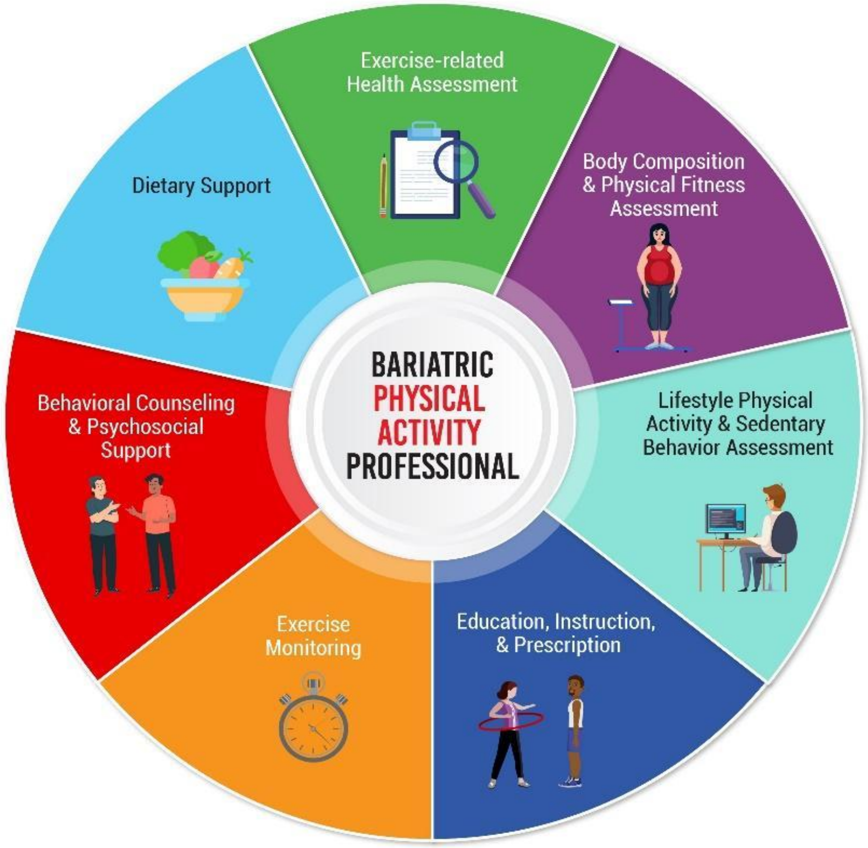

Supplement 11. Illustrated final model (Version A) for clinical use.

Image by Osama Alowaish, M.S., Teachers College – Columbia University, NY, NY

Copyright – The Authors (Stults-Kolehmainen et al., 2023). Use of figure permitted with Authors permission.
